# Supplementary material for: Optogenetics inspired transition metal dichalcogenide neuristors for in-memory deep recurrent neural networks
Source: Nat Commun. 2020 Jun 25;11:3211. doi: 10.1038/s41467-020-16985-0 (PMC7316775; doi:10.1038/s41467-020-16985-0)
Supplement: Supplementary file 1 — Supplementary Information [file 41467_2020_16985_MOESM1_ESM.pdf]

Supplementary Information

Optogenetics Inspired Transition Metal Dichalcogenide Neuristors for In-Memory Deep Recurrent Neural Networks

John et al.

Supplementary Note-1. Physical and DC-bias Characterization of photo-excitable neuristors (PENs)

An optoelectronic neuromorphic architecture calls for the need of atomically thin low-bandgap semiconducting channels as active switching matrices that would enable intimate optical and electrical control over the charge carrier concentration and conductance transitions to functionally mimic the role played by rhodopsins in optogenetics. 2D transition metal dichalcogenides (TMDCs) offer several attractive features in this regard with their atomic-level scalability, tunable bandgap and opto-electronically modulatable carrier concentrations[1]. Very recently, we demonstrated light-induced spike-timing dependent plasticity (STDP)-based weight modulations in MoS_2 neuristors, but with limited (12) number of states, symmetricity and plasticity[2]. Extending this concept to other semiconducting platforms, we demonstrate photo-excitable neuristors (PENs) based on Rhenium Disulfide (ReS_2) as optogenetic actuators modulating neural excitation and synaptic plasticity with optical stimuli. Designed in a field-effect configuration, the PENs harness the gate-controlled memresistance of the semiconducting channel, modulated additionally via photon pulses. The device fabrication and physical characterizations are described below (Supplementary Figure-1).

A scotch-tape method was used to exfoliate ReS_2 flakes from bulk crystal and was transferred onto a degenerately doped Si substrate with 285 nm SiO_2. The electrodes were patterned via photolithography, followed by thermal evaporation of Cr/Au (5/50 nm) and subsequent lift-off process (Supplementary Figure-1a). Supplementary Figures-1b-d shows the atomic force microscopy (AFM) and optical images of a ReS_2 sample. Height profile of the sample along the red line indicated the sample thickness (~5 nm). Raman characterization was performed to confirm the purity of the sample as shown in Supplementary Figure-1e. The ReS_2 PENs depicted a typical n-type depletion-operation with a linear mobility of 26 $cm^2V^{-1}s^{-1}$ (Supplementary Figures-1f-g). The field-effect mobility was estimated from the linear region in the I_d-V_g curve (V_g from 10 V to 40 V) using the equation $\mu = \frac{\mathrm{d}I_d}{\mathrm{d}V_g} \times \frac{L}{WC_iV_d}$, where L is the channel length, W is the channel width and C_i is the capacitance between the channel and the back gate per unit area ($C_i = \varepsilon_0 \varepsilon_r / d$; ε_0 is the vacuum permittivity, ε_r is the relative permittivity, and d is the thickness of SiO_2 layer), respectively. The sub-threshold swing was estimated to be ~2.5 V/dec, while the on-off ratio was greater than 10^6 for a drain voltage (V_{ds}) of 0.1 V. The channel length and width of the device was ~9 µm and ~20 µm respectively,

and thickness of the ReS_2 flake was ~5 nm. The devices depicted a clockwise hysteresis indicating electron capture (trapping) and release of these trapped electrons (detrapping) in the forward and reverse voltage scans[3].

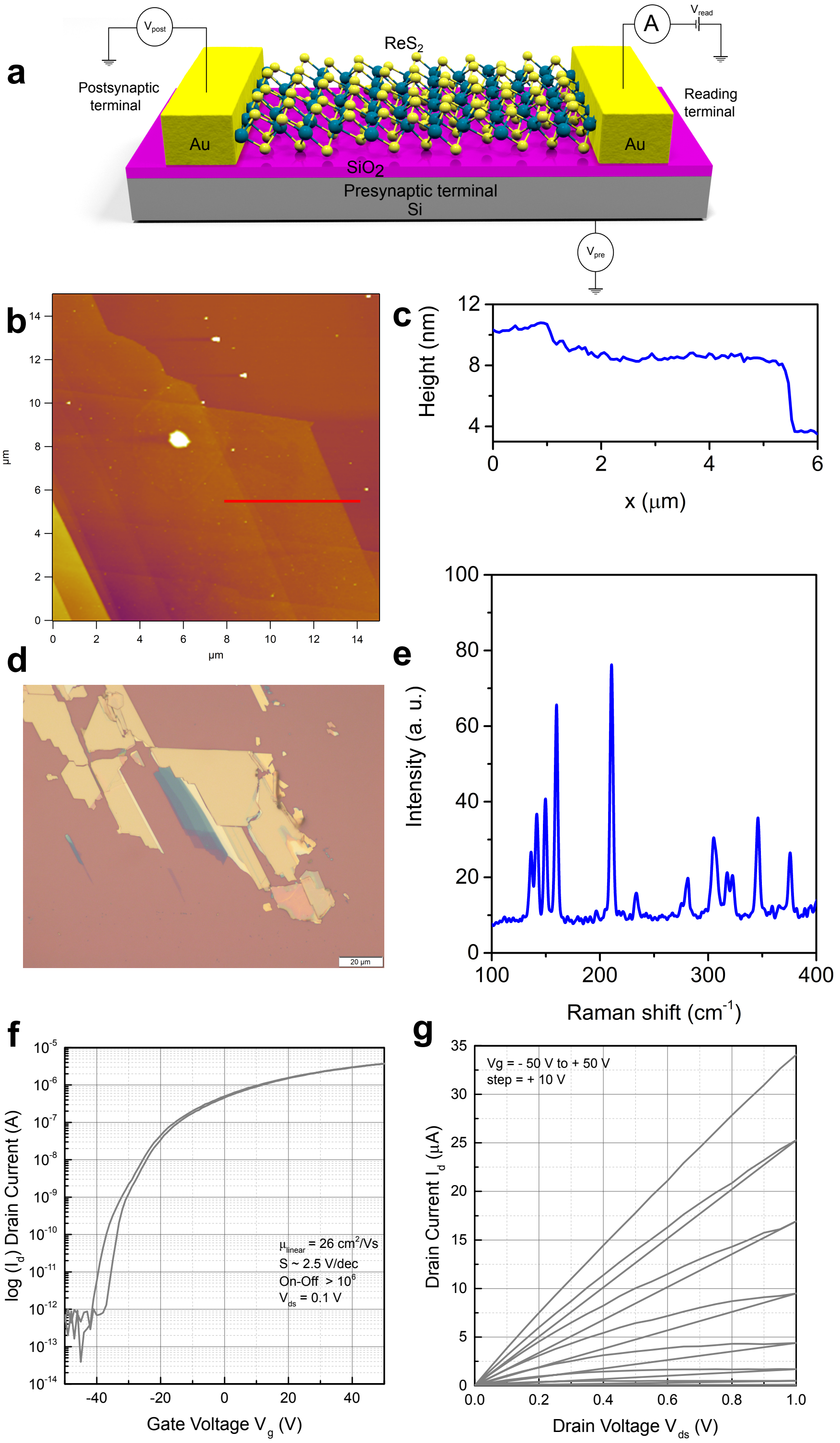

Supplementary Figure 1. Physical and DC-bias Characterization of 3-terminal PENs. a Device architecture. **b-d** AFM and optical images of a ReS_2 sample, respectively. **c** Height profile of ReS_2 flake along the red line in **b**, depicting a thickness of ~ 5 nm. **e** Raman spectra of ReS_2. **f-g** Transfer and Output characteristics of ReS_2 neuristors.

Supplementary Note-2. Designing Photo-sensitive Neurons with PENs

Using the PEN as a photo-activated ion-channel, an integrate and fire neuron (I&F) circuit was constructed (Figure-1b) using two operational amplifiers (op-amps). The first op-amp U1 acts as a leaky integrator (due to R_1 and C_1) for the input current and produces at its output a voltage equivalent to the neuronal membrane potential. Without the op-amp U_2 and the reset circuits, the steady state voltage at the output of U_1, $V_{mem,ss} = V_{ref} - R_1 G_{nr}(V_{in} - V_{ref})$. For proper operation of the circuit, V_{ref} and V_{thr} are chosen such that the following condition holds true:

$$V_{mem,ss} < V_{thr} < V_{ref}$$

Also, for a nominal positive value of I_{in}, we need $V_{in} > V_{ref}$. For regular operation, assume that the membrane potential V_{mem} starts from an initial value of V_{ref}. Due to integration of I_{in}, V_{mem} keeps reducing towards its steady state value $V_{mem,ss}$. The moment V_{mem} falls below V_{thr}, the output of op-amp U_2 (acting as a comparator) produces a sharp transition at its output similar to the upswing of a neuronal action potential due to sodium channel activation. This disables the switch S_2 and C_2 starts charging through the resistor R_2. The rapid change in voltage at the output of U_2 is similar to the upstroke of the action potential. The time constant set by R_2C_2 and the voltage V_1 determine the on time (T_{on}) of the action potential at V_{spk} as shown in Supplementary Equation-1. The charging of V_{spk} has an effect similar to the slow activation of hyperpolarizing potassium channels—it eventually turns ON the switch S1, resetting V_{mem} to its resting potential. For proper operation of the reset mode, it is required that V_1 be larger than the threshold voltage (V_{thsw}) of the switch S_1, i.e. $V_1 > V_{thsw}$. Once the voltage of V_{spk} reaches V_{thsw}, it discharges the capacitor C_1 and resets the membrane voltage V_{mem} to V_{ref} . This operation is similar to the hyperpolarization of the membrane due to opening of potassium ion channels in a biological neuron. Since the reset voltage at V_{mem} is higher than its steady state value, it starts reducing again and the cycle continues. The off duration (T_{off}) of V_{spk} can be approximated by Supplementary Equation-2 provided $T_{off} < R_1C_1$. Values of R_1, R_2, C_1, C_2, and V_{thr} are chosen such that time period, $T_{on} + T_{off} \approx T_{off}$.

$$T_{on} = R_2C_2 \ln\left(\frac{V_1}{V_1 - V_{thsw}}\right) \ldots\ldots\ldots\ldots\ldots\ldots\ldots\ldots (1)$$

$$T_{off} = \frac{(V_{ref} - V_{thr})C}{(V_{in} - V_{ref})G_{nr}} \ldots\ldots\ldots\ldots\ldots\ldots\ldots\ldots\ldots\ldots (2)$$

Supplementary Table-1 reports the values of the components and voltage sources used in this experiment.

Supplementary Table 1. Values of the components used in the neuron circuit.

Parameter	Value	Unit
R_1	150	kΩ
R_2	100	kΩ
G_{nr}	6.66	μS
C_1	10	nF
C_2	1	nF
V_{in}	5	V
V_{ref}	4	V
V_{thr}	3.6	V
V_1	5	V
V_{thsw}	2.4	V

The top half of Figure-1b (main text) depicts the frequency modulated pulse train, V_{spk}, in the room light and with a blue light source using the PEN as a conductance input. The reversal potential V_{in} of the PEN is set such the neuron produces a nominal firing rate without any illumination. On shining blue light (λ=445 nm, 65 mWcm^{-2}) on the PEN for 10 sec, the conductance G_{nr} increased leading to an increase in the input current, resulting in higher firing rate (time period reduces by 39 %, from 935 μs to 570 μs) of the neuron (top-Figure-1b) governed by the parameters of the circuit (S1). On the other hand, the same neuron when connected to a standard resistor (200 kΩ) did not show any appreciable change in frequency under the same illumination condition (bottom-Figure-1b). This selective ability to address neurons with optical pulses, a hallmark of optogenetics, is hitherto not demonstrated for neuromorphic circuits.

Supplementary Note-3. PENs as Photo-sensitive Synapses

Learning in the brain occurs via strengthening and weakening of synaptic connections, interconnecting a myriad of neurons[4,5]. This modification of synaptic strength, referred to as weight plasticity, is responsible for spatial memory storage and forms the basis of learning mechanisms[6,7]. A spike-based formulation of Hebbian learning, spike-timing dependent plasticity (STDP) is considered to be the first law of synaptic plasticity and underlies the basis

of associative learning across several species from locusts to fishes to rats to humans[4,8,9]. Memory traces of past experiences are encoded as temporal correlations between electrical pre- and post-synaptic neurons, resulting in a temporally asymmetric form of Hebbian learning. Temporal correlations between pre- and postsynaptic spikes defines the sign and magnitude of long-term synaptic modifications, referred to as the STDP function or learning window, and varies tremendously across synapse types, dendritic compartments and brain regions[8,10].

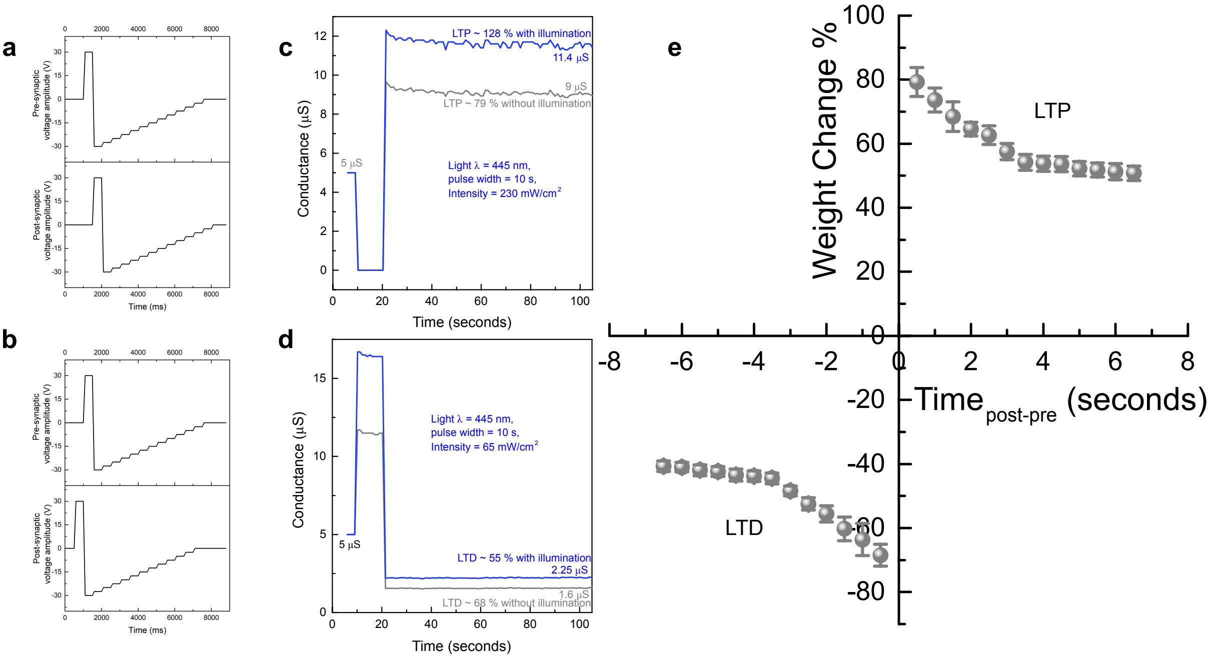

Supplementary Figure 2. Electrical asymmetric Hebbian STDP rules in ReS_2 neuristors. a-b Input waveforms used for STDP measurements. **c-d** Conductance variations corresponding to LTP and LTD measured without and with optical illumination. **e** STDP: Long-term weight changes as a function of the interval between pre- and post-synaptic spikes ($Time_{post\text{-}pre}$) (dark).

Repeated arrival of pre-post or post-pre spike pairs translated into time-integrated voltage amplitude differences between the pre- and post-synaptic neurons (V_{pre}–V_{post}), which when crossed the threshold for non-volatile changes, induced long-term potentiation (LTP)/ depression (LTD) in the channel conductance[11,12] (Supplementary Figures-2a-d). The conductance was monitored before, during and after application of the spike pairs, and was recorded as a function of the pulse interval between pre- and postsynaptic spikes (Supplementary Figure-2e). Weight changes were predominant at small pulse intervals, and weakened with increase in the interval, reflecting strong temporal correlations between the pre- and postsynaptic spikes. The resultant weight changes depended on the net effective voltage developed across the device integrated over a time t[11,13]. For example, when spike pairs corresponding to Supplementary Figure-2a was applied to the device at an interval ($t_{post\text{-}pre}$) of

+500 ms, the maximum net voltage developed across the device was V_{pre}-V_{post} = (-30) – (+30) = -60 V and this resulted in a permanent increase in the channel conductance or LTP (~79 %) as depicted by Supplementary Figure-2c. On arrival of presynaptic pulses after postsynaptic pulses, i.e. $t_{post-pre}$ of -500. ms (Supplementary Figure-2b), the maximum net voltage developed across the device was V_{pre}-V_{post} = (+30) – (-30) = +60 V and this resulted in a decrease in conductance or LTD (~68 %), as shown in Supplementary Figure-2d. These measurements were repeated for several combinations of spike intervals and the weight changes were plotted as a function of $t_{post-pre}$ as shown in Supplementary Figure-2e.

Photo-modulated STDP:

Illumination with red light (λ= 623 nm) induced long-term potentiation at all intensities, resulting in a positive shift of the STDP functions (Supplementary Figure-3a). The shift depicted a direct relationship with the intensity of optical excitation. Increasing the intensity of optical stimuli from 65 to 230 mWcm^{-2} resulted in an increased LTP from 83 to 117 % (for $t_{post-pre}$= +500 ms), in comparison to 79% for the pure electrical STDP. The shift also depicted a wavelength dependence as expected. Illumination with lower wavelengths (green, λ=525 nm and blue, λ= 445 nm) induced higher degree of manipulations of the STDP function due to the higher energy of irradiation as shown in Figure-1c, Supplementary Figures-3b-c. Supplementary Figure-3d compares this behaviour for all the three wavelengths used in this study. For the same intensity of illumination (65 mWcm^{-2}), blue illumination caused a 94 % LTP, while green and red illuminations accounted for 90 % and 83 % respectively.

With regards to the LTD window, all optical stimulations counteracted the electrical LTD effects. Such an interaction between the optical LTP and electrical LTD resulted in convoluted conductance/weight changes as shown in the Figure-1C, Supplementary Figure-3. This becomes particularly evident for the cross-over regions (marked by the dashed circle in the figures) when illuminated with the highest dosage of optical stimuli. Upon continuous illumination with red light (230 mWcm^{-2}), the devices depicted a net LTD for $t_{post-pre}$ upto 2000 ms, indicating dominance of the electrical LTD over the photo-modulation effects. For intervals beyond 2000 ms, the effects of electrical LTD weakened, resulting in a net LTP (Figure-1c). For the highest photo-dose of green and blue light, the photo-modulated LTP completely compensated the electrical-LTD at all spike intervals, resulting in potentiation irrespective of the temporal correlations between the electrical pre and post-synaptic spikes

(Figure-1c). Removal of the optical stimulation resulted in retrieval of the original STDP function after 2 hours as shown in Supplementary Figure-4.

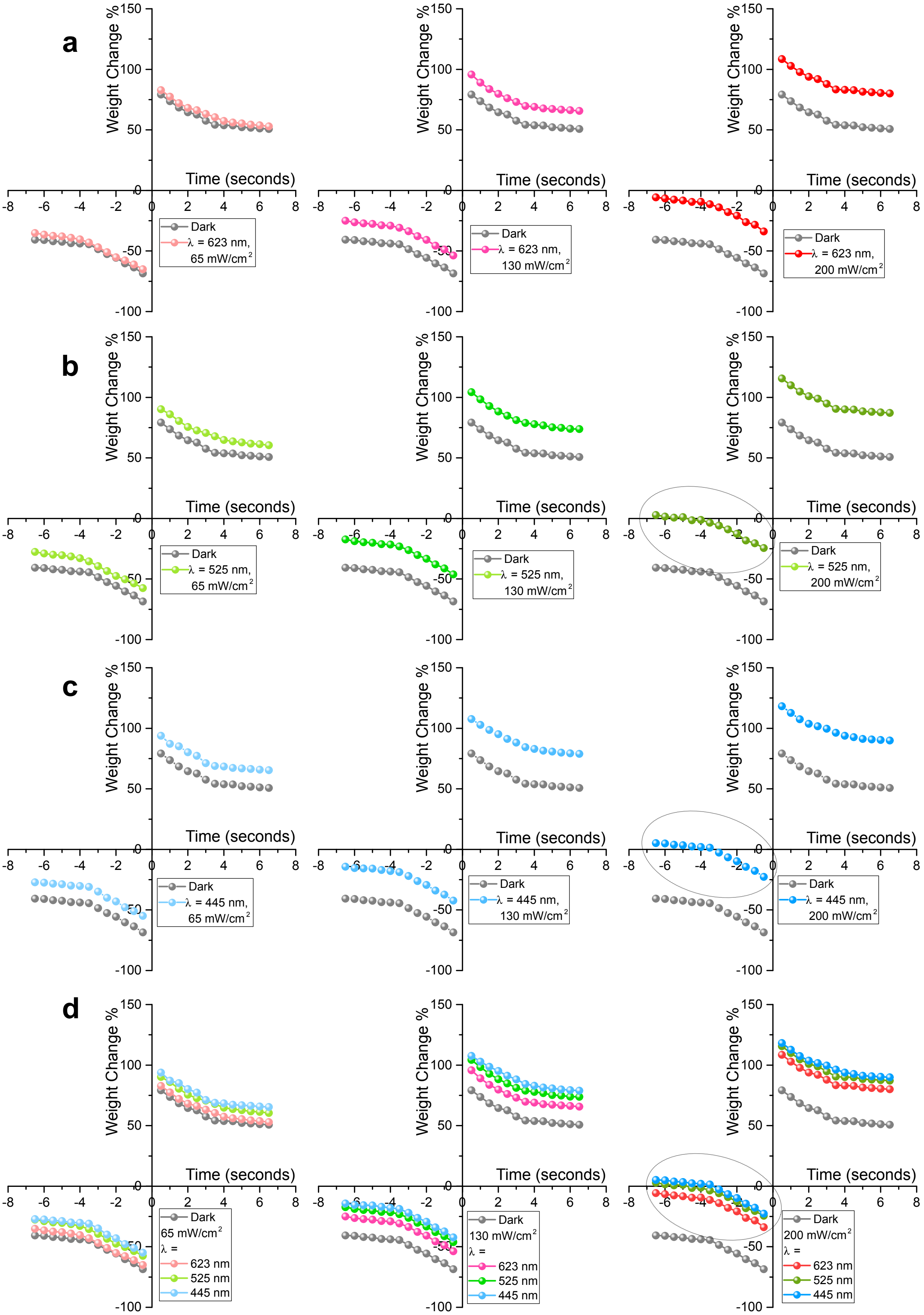

Supplementary Figure 3. Photo-modulated STDP learning rules in PENs. The devices were subjected to continuous optical stimulations during the electrical STDP measurements. This resulted in a modulation of the STDP window as a function of the wavelength and intensity of photo-dosage and exposure, akin to optogenetic measurements. The graphs (read row-wise) depict modulations of STDP window upon illumination with **a** red (623 nm), **b** green (525 nm) and **c** blue (445 nm) light, as a function of the intensity of photo-dosage. **d** compares the modulations as a function of the wavelength of illumination for intensities 65, 130 and 200 mWcm^{-2}.

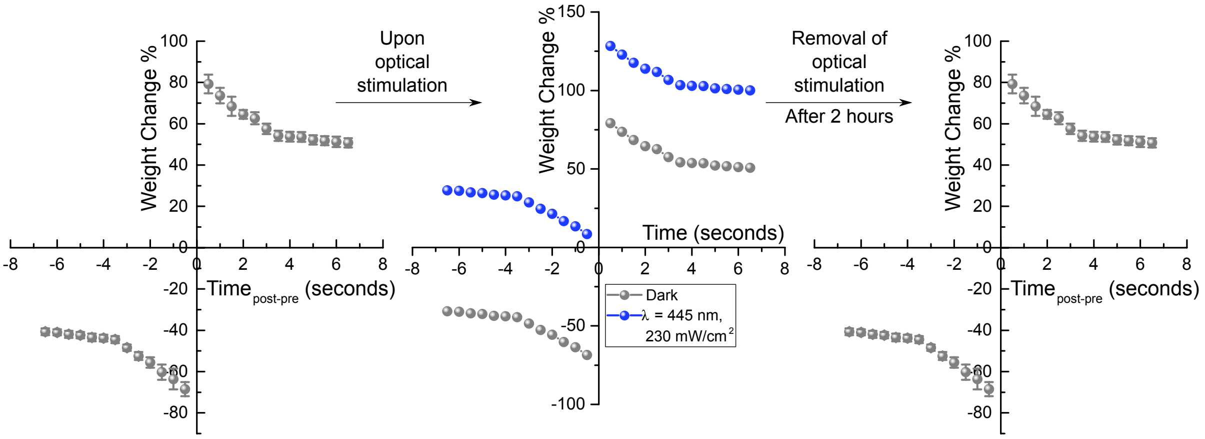

Supplementary Figure 4. Recovery of the original electrical STDP function after photomodulation. Removal of the optical stimulation resulted in retrieval of the original STDP function after 2 hours as shown above, indicating retention of the photo-modulated states to be ~2 hours for our best-performing devices. The average retention was observed to be ~900 seconds for the condition of no crossover between adjacent states, acceptable for training[14].

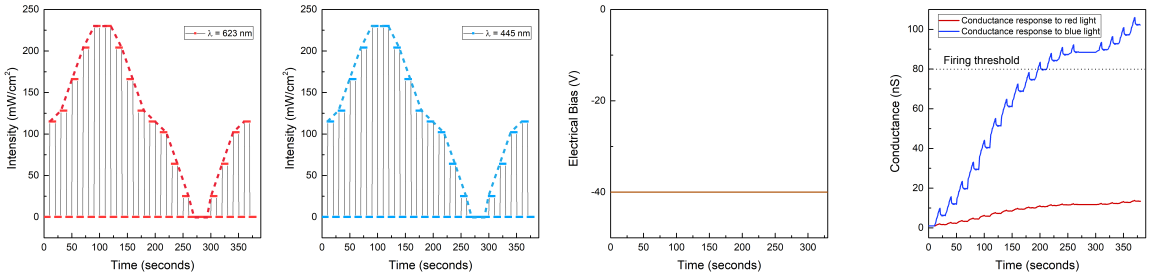

Supplementary Figure 5. Spatiotemporally-selective activation of synapses. Application of sinusoidal global optical clock signals to the PENs resulted in a long-term weight modulation as depicted by the graph on the right. A constant electrical bias of -40 V was applied during these measurements to set the initial conductance state to a low value of ~1 nS. We demarcate a firing threshold just to highlight the fact that the difference in photoresponse or wavelength selectivity of our artificial synapses would allow a wide flexibility for setting the threshold for neuronal firing. In other words, we foresee development of artificial synapses that respond to narrow bands of optical pulses in future and would like to point out that our approach would enable setting of desired pre-set thresholds for selective neuronal firing when such artificial synapses are connected to artificial neurons.

Supplementary Note-4. Optically-addressable multi-level memory for DRNNs

To demonstrate the extremely high density of non-volatile states available for analog computation, the PENs were subjected to an input optical pulse train of constant pulse width and interval (10 s, λ= 623 nm (Supplementary Figure-6), 525 nm (Supplementary Figure-7) and 445 nm (Supplementary Figure-8), 65 mWcm^{-2} intensity). A constant electrical bias of -40 V was applied at the gate terminal during these measurements to maximize the linearity of weight updates. The sub-plots **a-t** in each figure depicts the magnified view of ~50 non-volatile states, totalling to ~980 states.

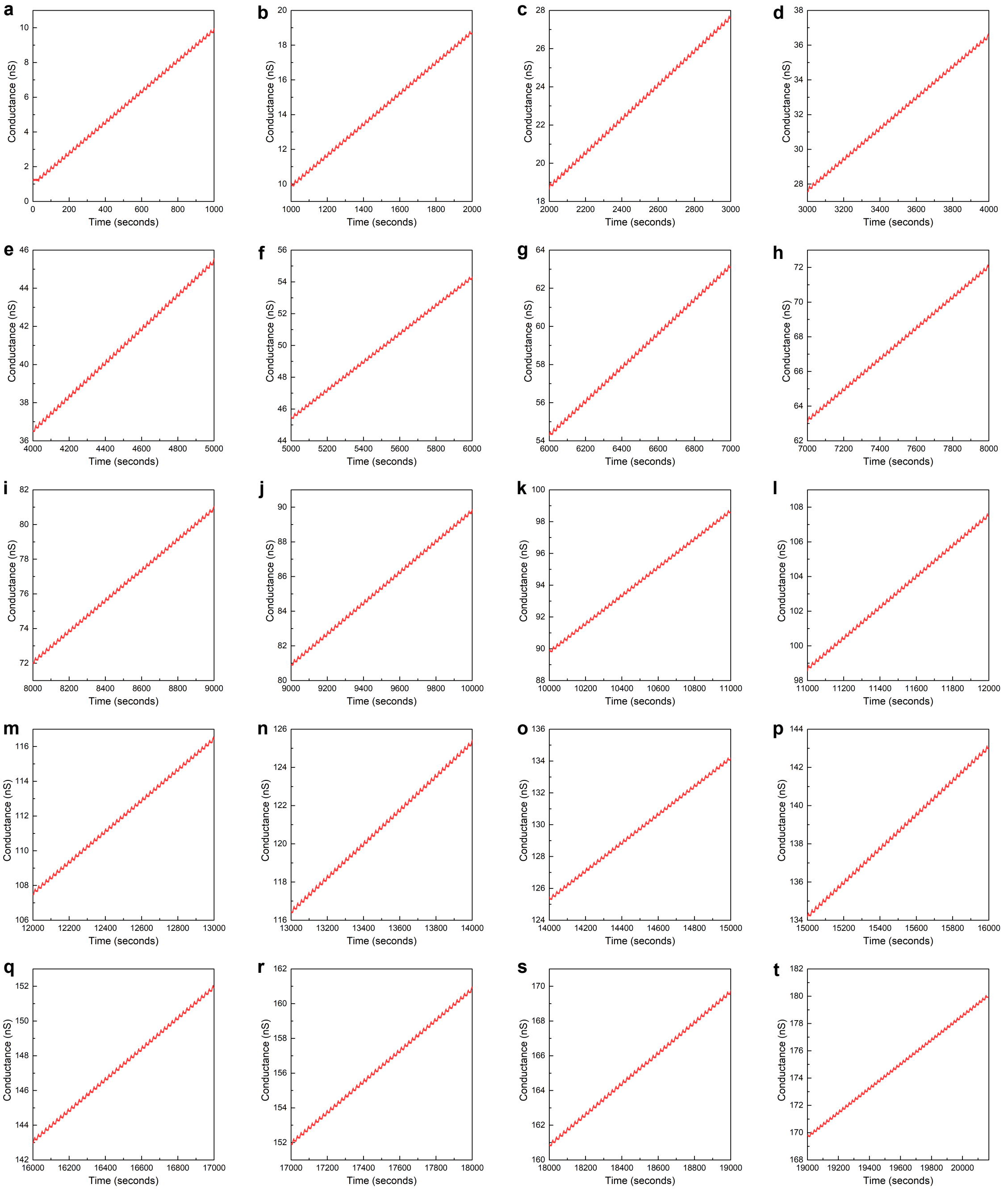

Supplementary Figure 6. Optically addressed multi-level memory for DRNNs. Activation with red light (10s, λ= 623 nm, 65 mWcm^{-2} intensity). **a-t** Each graph depicts ~50 non-volatile states, with a linear step size ~0.18 nS spanning from 1.25 to 180 nS.

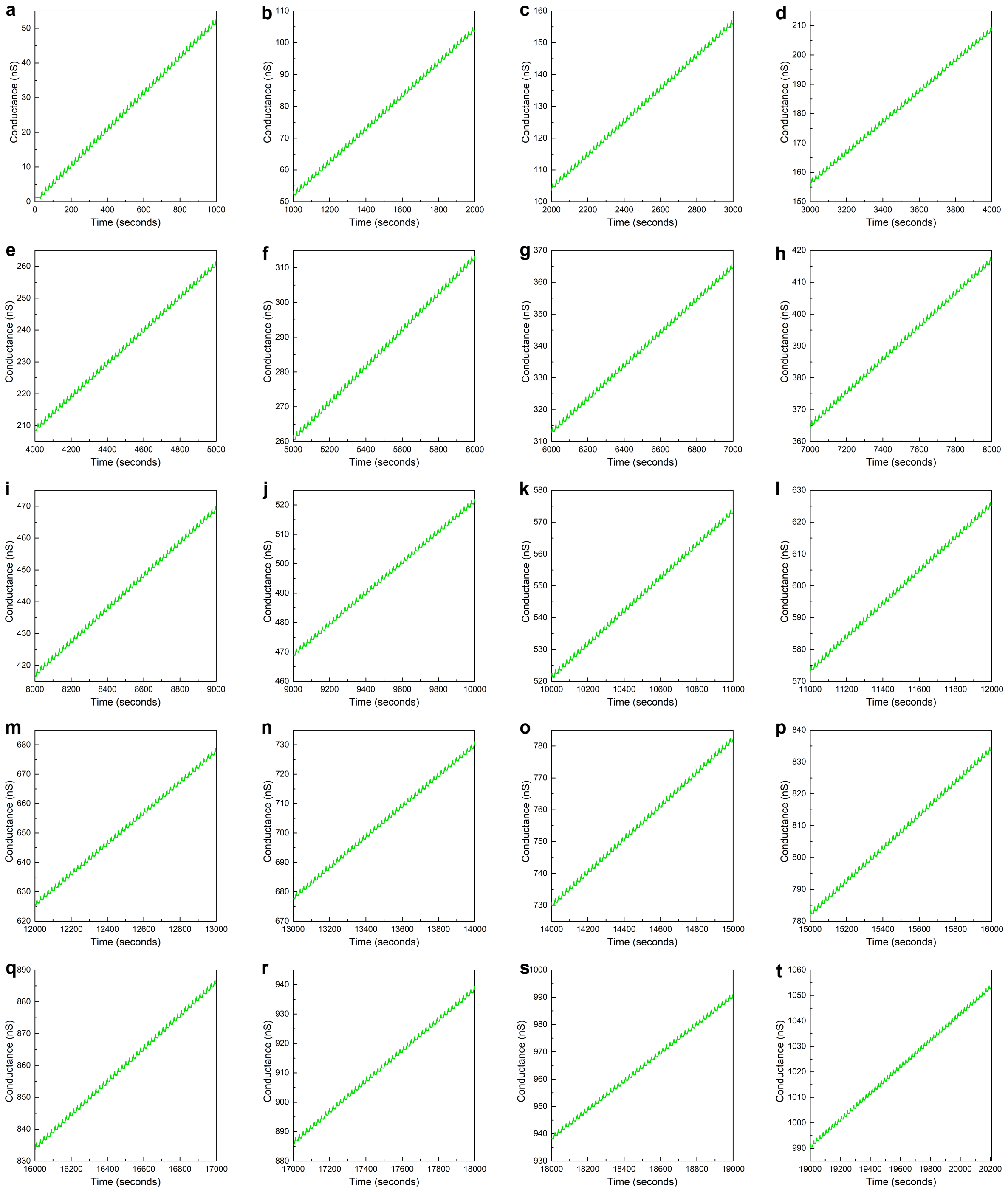

Supplementary Figure 7. Optically addressed multi-level memory for DRNNs. Activation with green light (10 s, λ= 525 nm, 65 mWcm^{-2} intensity). **a-t** Each graph depicts ~50 non-volatile states, with a linear step size ~1.07 nS spanning from 1.25 nS to 1.05 µS.

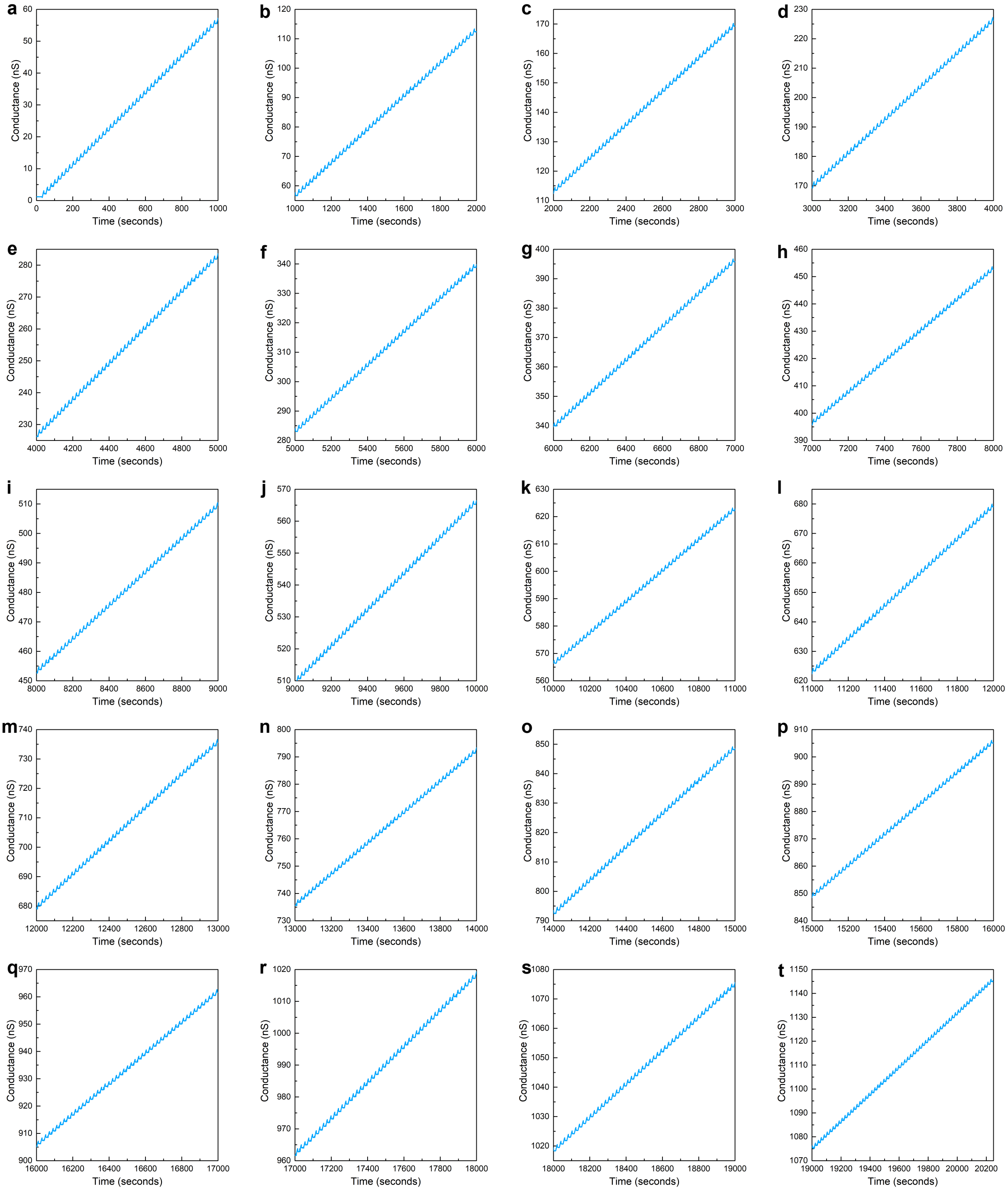

Supplementary Figure 8. Optically addressed multi-level memory for DRNNs. Activation with blue light (10 s, λ= 445 nm, 65 $mWcm^{-2}$ intensity). **a-t** Each graph depicts ~50 non-volatile states, with a linear step size ~1.16 nS spanning from 1.16 nS to 1.15 µS.

Supplementary Note-5. Optical Write and Electrical Erase Operations

Stability of the individual states, endurance and dynamic range becomes cardinal evaluation parameters in the case of such multilevel memories, since the on-off ratio between the continuously modulated conductance states lie close to each other[15]. A higher dynamic range would ensure improved device reliability, increased number of distinctly addressable states would improve the memory storage capabilities, while excellent endurance properties would ensure stable device operation[16,17]. Supplementary Figure-9 shows a magnified view of the conductance response during the first 128 voltage-controlled cycling tests, limits of linear weight updates, and Supplementary Figure-10 depicts the endurance characteristics of the non-volatile states.

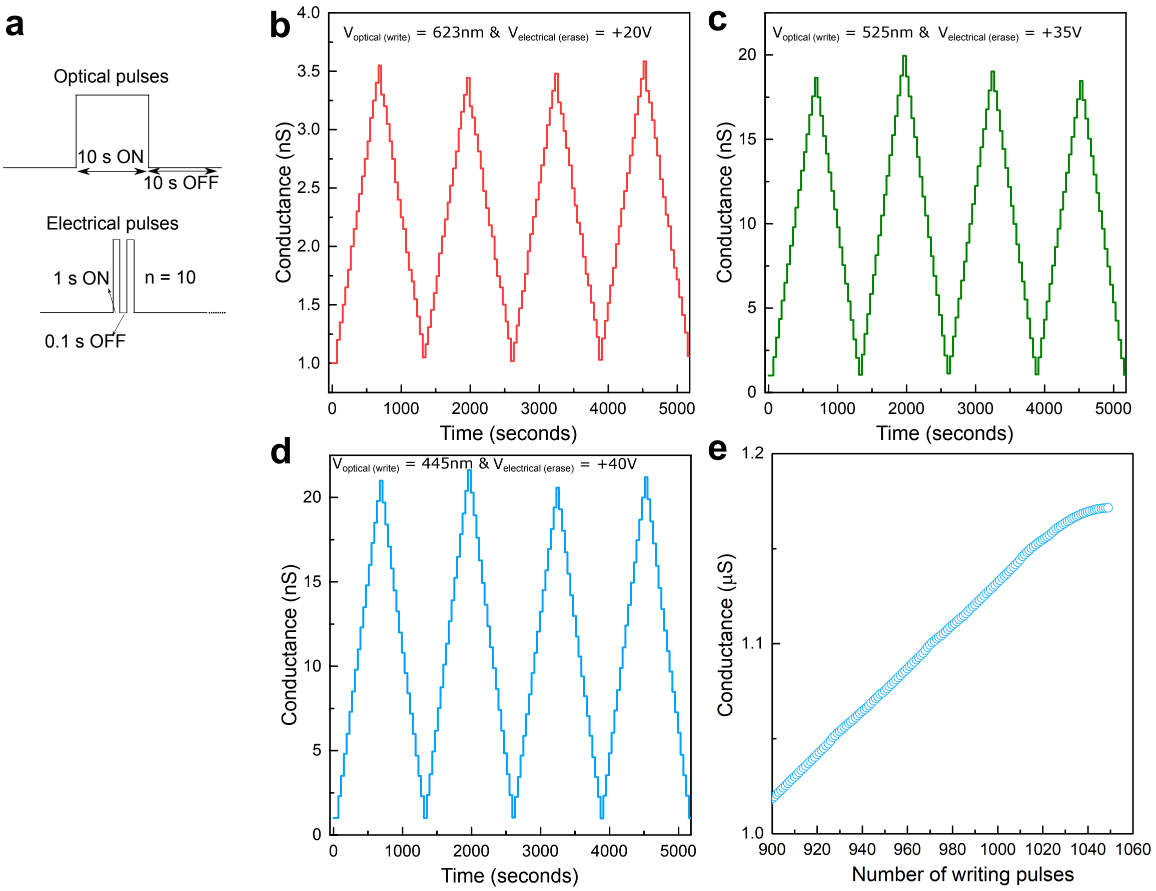

Supplementary Figure 9. Optical Write and Electrical Erase Operations. Application of synergistic optoelectronic pulses **a** result in precisely controlled near-symmetric bidirectional weight changes via optical potentiation and electrical depression as shown in **b-d**. **b-d** shows a magnified view of the conductance response during the first 128 voltage-controlled cycling tests. **e** To test the limits of linear weight updates, the device conductance was monitored till saturation. We observe that the linear weight updates continue up to ~1018 optical writing pulses for blue light illumination (65 $mWcm^{-2}$) after which the update step size gradually ceases. We clarify that these limits may vary from device to device. The value reported in the manuscript (980 addressable states) is an average obtained by measuring 5 devices repeatedly across 3 wavelengths of illumination. Moreover,

from an algorithm perspective, the exact saturation level is not important as long as the dynamic range is high enough. The 2-shot writing scheme we adopt (explained in Supplementary Note-8 [see below]) takes into account the more critical variation in the slope of the curve to eliminate any sort of mismatches at the network level.

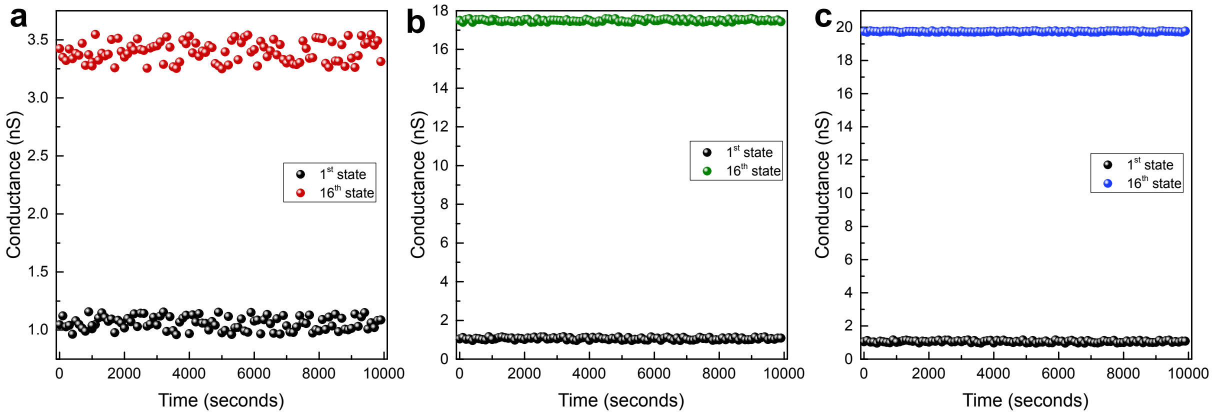

Supplementary Figure 10. Endurance of the non-volatile states. The devices were switched between the 1st and 16th conductance state by appropriate optical write and electrical erase operations and the conductance states were read by a reading voltage of 0.1 V. The PENs depicted excellent endurance characteristics (tested up 10^4 seconds) for all the three wavelengths of optical stimulation, namely **a** red, **b** green and **c** blue.

Supplementary Note-6. Optoelectronic abacus operations.

Multiplication of ‘7x3’ was implemented as successive additions as explained in the main text (Supplementary Figure-11a). Carry over operations included appropriate electrical RESET operations at the PEN representing the unit’s place and concurrent optical update operations at the ten’s place, resulting in a correct product ‘21’. For the subtraction operation of ’27-14’ (Supplementary Figure-11b), the PENs were initially programmed to represent the minuend (‘7’), before proceeding with appropriately designed electrical erase pulses corresponding to the subtrahend (‘4’), to get the final result ‘3’ in the unit’s place. Similar operations at the ten’s place yielded ‘1’. The conductance states were read by 0.1 V to get the final correct result ‘13’. For the division operation ‘15/5’, a third PEN was programmed as a counter (green beads) with appropriate optical updates for each complete subtraction of the divisor ‘5’ from the dividend ‘15’, to get the final quotient of ‘3’ as shown in Supplementary Figure-11c. The carryover, counter update operations and control of the light sources were carried out via an external microcontroller in the current experimental setup. However, for real-life practical implementations, all these operations could be carried out via on-chip peripheral circuitry. Most importantly, all arithmetic calculations and data storage were carried out simultaneously in the three PENs employed here, elementarily demonstrating their in-memory computing capability.

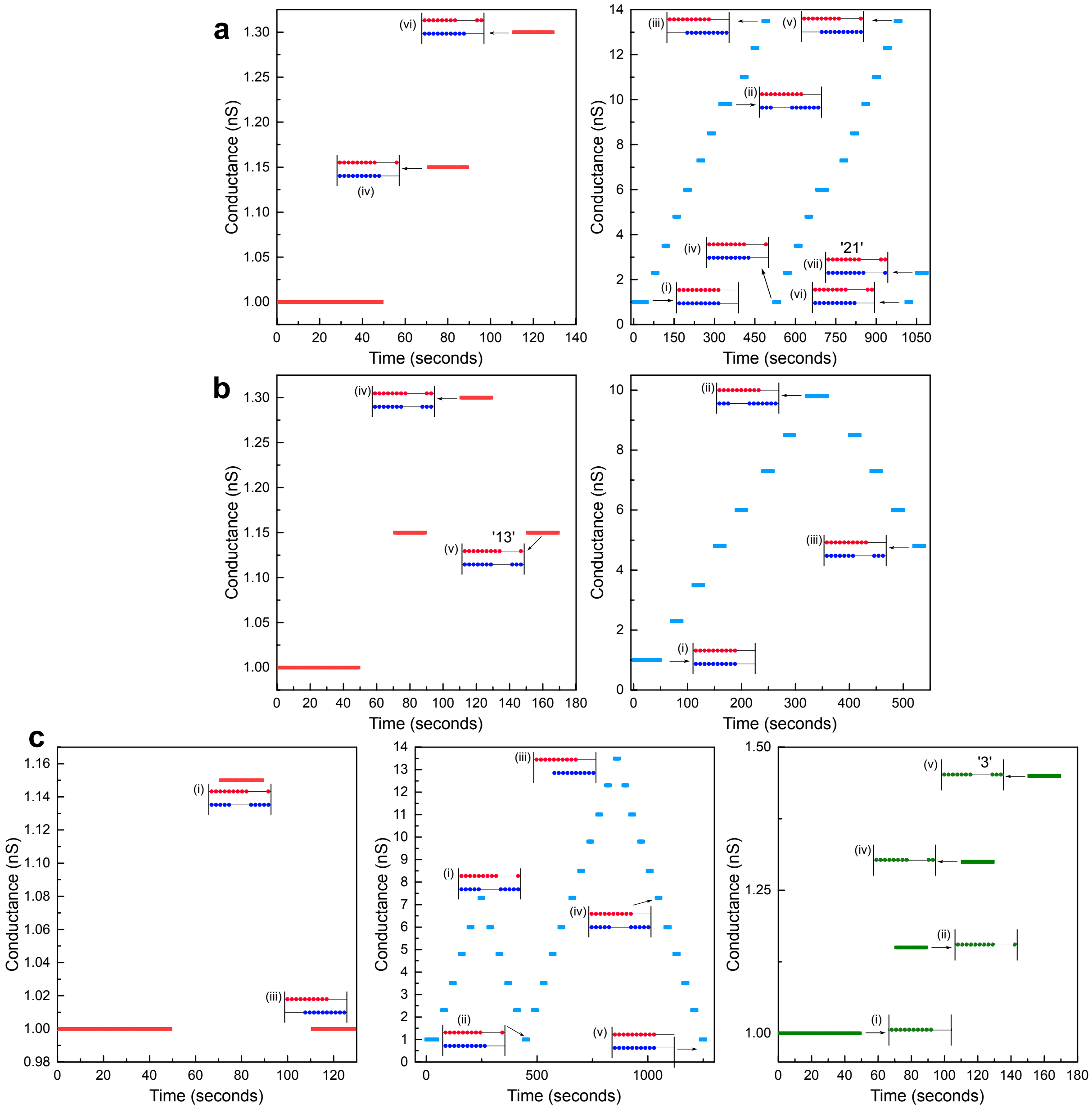

Supplementary Figure 11. Optoelectronic Abacus Operation. Precise optical potentiation and electrical depression enabled facile emulation of arithmetic operations, analogous to an abacus. Multiple PENs were employed to represent the unit's (blue bead), ten's (red bead) and counter's (green bead) place and programming steps were designed as per the arithmetic operation under calculation. Optical stimulations resulted in potentiation represented by the rightward sliding of the beads, while electrical stimulations caused depression represented leftward sliding of the beads. The modus operandi of the **a** multiplication, **b** subtraction and **c** division operations are indicated with necessary illustrations as insets.

Supplementary Note-7. Mechanistic Understanding

Nature of defects:

The exact nature of traps or defects in a material system is dependent on the composition and the fabrication processes involved. In transition metal dichalcogenides, studies have indicated different possible origins for the electron trapping and detrapping mechanisms, including surface adsorbates[18–20], electron trapping at the semiconductor-dielectric interface[21,22] and intrinsic semiconductor defects/lattice defects[23,24]. Since our measurements were performed in high-vacuum conditions, the probability of surface adsorbates are highly reduced. Thus, we attribute the origin of trapping-detrapping mechanism in our measurements to defects in the semiconductor itself or/and the at the semiconductor-dielectric interface. The high value of subthreshold slope (~2.5 V/dec) in our devices support the presence of traps influencing the ReS_2 channel in accordance with similar measurements in literature[25,26]. In the present work, we utilize a combination of optical and electrical pulses to fill and empty these traps and to induce non-volatile conductance changes in our field-effect transistors (FETs). The number of distinct conductance states are determined primarily by the programming pulse resolution and recombination kinetics of the photo-generated carriers, and hence, the programming pulses could be optimized to achieve a very good linearity.

Programming scheme to maximize linearity:

To program states in a linear manner, we need to carefully select the gate voltage, the initial conductance state, the drain voltage and intensity of light illumination as explained below.

Firstly, to maximize linearity in write and erase, we apply and maintain a gate bias (-40 V) to our devices to take it to their depleted-state before starting our measurements (Supplementary Note-3 Figure-5). The initial low conductance is a critical step in achieving linearity, as also reported by other investigations[14,27]. By constantly biasing the devices at a gate voltage that depletes the majority carriers in the system (-40 V in the case of ReS_2 devices or negative V_{gs} for any n-type semiconductor), we ensure that the traps are empty and the background carrier concentration is minimized[28], and this also increases the probability of photocarrier trapping[26]. Upon illumination, the photogenerated carriers fill up these traps, resulting in a permanent increase in the channel conductance due to PPC (also termed photogating in literature)[25]. Finally, the V_{ds} should be high enough to overcome possible contact resistance effects and should result in an I_{ds} which is significantly larger than I_{gs}, necessary to ensure accurate weight update readouts.

In summary, a V_{gs} that depletes the major carriers and keeps the trap states empty, a V_{ds} that ensures the channel currents are much larger that the gate leakage currents, a low initial conductance/background carrier concentration to avoid interference with the photogenerated carriers and an appropriate light intensity to generate photocarriers which can respond to the applied V_{gs} to fill the traps should give the highest chance of linearity (Supplementary Figure-12).

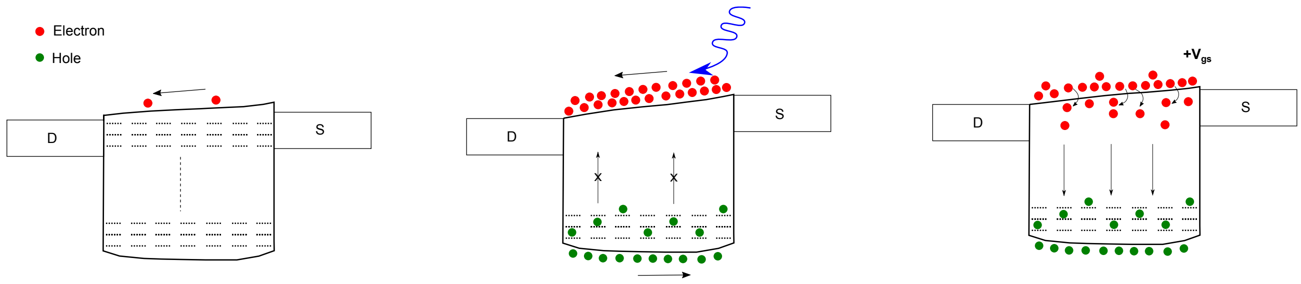

1. READ: Initially the trap states are emptied by the $-V_{gs}$.

2. WRITE: Photo-carriers generated upon Illumination populates the channel (increases current), but gets trapped in the bulk of the semiconductor or at the semiconductor-dielectric interface, resulting in persistence photoconductivity (PPC).

3. ERASE: + V_{gs} raises the Fermi level to induce electron accumulation in the channel and accelerates the recombination of electrons with the holes in the trap sites decreasing the conductance.

Supplementary Figure 12. Mechanistic understanding of the Write-Erase process.

Effect of constant gate bias V_{gs}:

We first show that the linearity is modulated as a function of the applied constant gate bias during optical potentiation for ReS_2 FETs. Supplementary Figure-13 below shows the weight updates: optical potentiation with different constant gate biases, and electrical depression. As can be seen, maintaining a large negative V_{gs} that depletes the majority carriers in the system allows us to write weights more linearly when compared to partially depleted (~0 V) and accumulative (high $+V_{gs}$) voltages for ReS_2 FETs. As explained above, we believe this negative V_{gs} helps empty the traps, allowing us to control the photo-generated carriers more precisely, resulting in linear weight updates[28]. Since we perturb the carrier concentration in small steps, the erasing is also fairly linear compared to the very high conductances reached at zero/positive V_{gs}. At zero/positive V_{gs}, our FET is already ON with a high concentration of background carrier in the channel (Supplementary Figure-1f), which screens the effect of photo-generated carriers, resulting in lower magnitude and non-linear update of weights during potentiation. The larger value of absolute conductance also results in uncontrollable (non-linear) erase steps, sometimes even going below the level of initial conductance. The data presented in the above graph represent conclusions from 15 ReS_2 FETs.

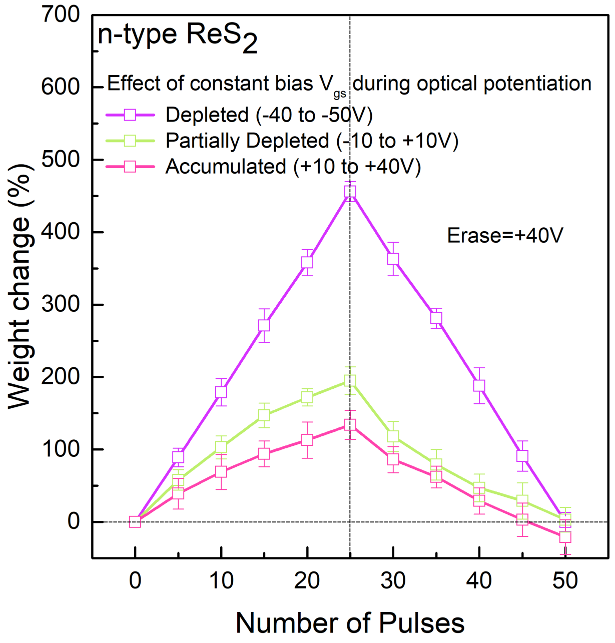

Supplementary Figure 13. Weight changes in ReS_2 PENs [Data from 15 devices] as a function of the constant bias V_{gs}. For potentiation, blue light pulses of λ=445 nm and intensity=65 $mWcm^{-2}$ was used with a pulse width and interval of 10 s each. Key: Depleted- refers to a range of V_{gs} -40 to -50 V depending on the V_{on} of the respective PEN. Similarly, Partially Depleted- refers to a range of V_{gs} -10 to +10 V and Accumulated- refers to a range of V_{gs} +10 to +40 V. The graph represents our conclusion from experimental measurement of 15 devices. The error bars represent the variation among devices.

The effectiveness of the gate voltage in inducing write and erase linearity is also linked to the background conductance of the devices[28]. Medium and high initial conductance states result in lower magnitude of weight updates due to the screening effects of the background charge carrier concentration and the high absolute value of conductance results in uncontrollable non-linear asymmetric erasing (Supplementary Figure-14).

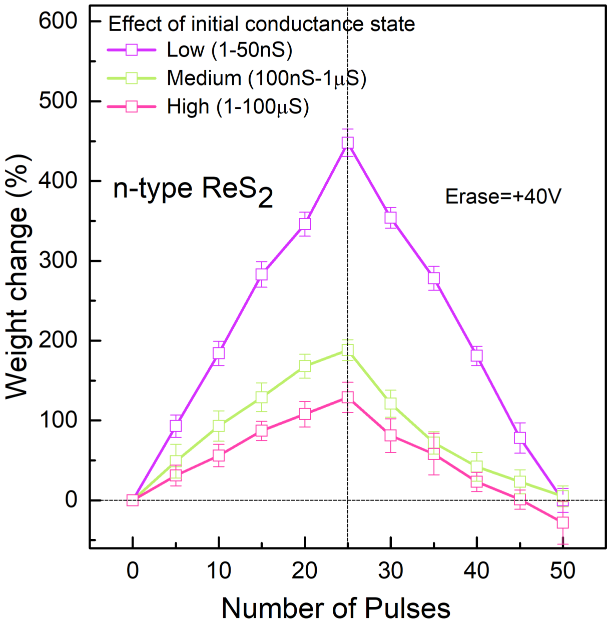

Supplementary Figure 14. Weight changes in ReS_2 [Data from 15 devices] PENs as a function of the initial conductance state. Key: Low- refers to a range of 1-50 nS depending on the respective PEN. Similarly, Medium- refers to a range 100 nS-1 µS and High- refers to a range of 1-100 µS. The initial conductance here refers to the conductance of the FET before potentiation and depression measurements. For a fair comparison, the same fully depleting voltage (V_{gs}=-40 V) was applied to all devices.

Effect of drain bias V_{ds}:

A V_{ds} that ensures the channel currents are much larger that the leakage currents is necessary for accurate weight read-outs. In our TMDC FETs, we typically apply a V_{ds} value of 0.1 V for our measurements. Lower applied drain voltages resulted in inconsistent results especially when the gate leakage currents (I_{gs}) were high. The effect of V_{ds} can be more clearly demonstrated in the P3HT system by comparing the linearity of weight change responses under the same optical pulsing conditions and V_{gs} bias. Please refer to Supplementary Note-9 Figure-25 for more details [see below].

Effect of optical illumination (Intensity):

We next show that the weight update steps could be further modulated by changing the intensity of illumination (Supplementary Figure-15). Higher optical intensities (consequently increased photocarrier trapping) results in increased weight changes, albeit with an increased spread.

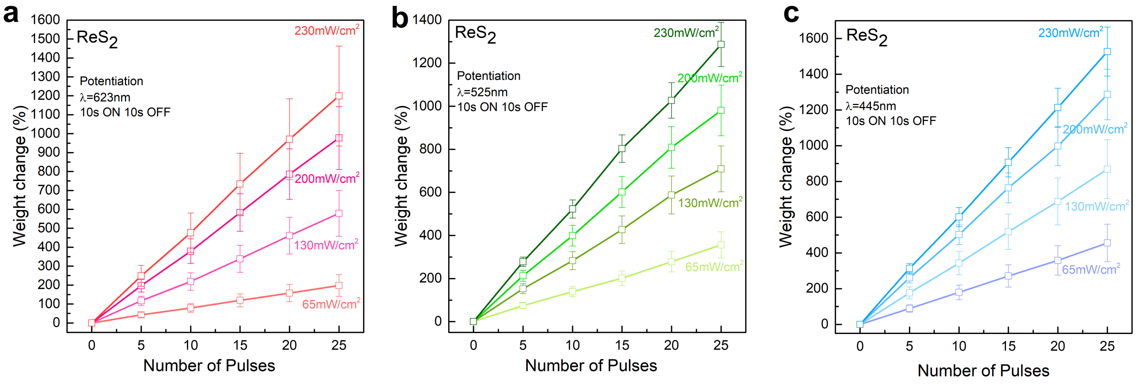

Supplementary Figure 15. Weight changes as a function of the light intensity for *λ*= **a** 623, **b** 525 and **c** 445nm, 10 s ON, 10 s OFF in ReS_2 PENs [Data from 5 devices]. The graph represents our conclusion from experimental measurement of 5 ReS_2 FETs. The error bars represent the variation among devices.

Linearity of electrical erase:

The erase process for n-type ReS_2 via application of a positive gate voltage raises the Fermi level to induce electron accumulation in the channel and accelerates the recombination of electrons with the holes in the trap sites resulting in lowered current levels as shown in Figures-3b-d and Supplementary Figure-9. This is in line with other investigations[25,29], where voltage pulses that induced majority carriers were used to eliminate persistent photocurrent. The linear nature of erase is made possible by carefully selecting the erase voltages after extensive optimization as shown below Supplementary Figure-16.

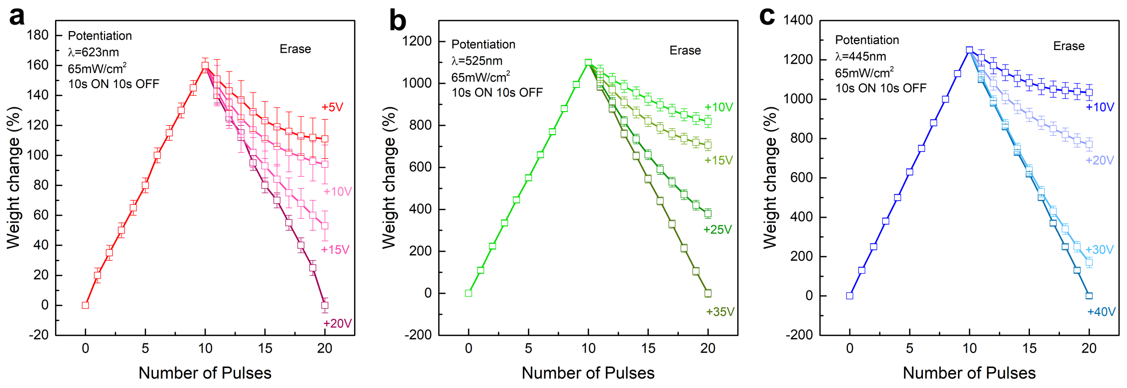

Supplementary Figure 16. Weight changes in ReS_2 PENs [Data from 15 devices] as a function of the erase voltages. a-c depicts the optimization of erase voltages as a function of the optical illumination wavelength. The graph represents our conclusion from experimental measurements of 15 ReS_2 FETs. The error bars represent the variation among devices.

Increasing the erase gate voltage raises the Fermi level further to accumulate more electrons in the channel which can recombine with the trapped holes, allowing us to tune our symmetry. This is similar to strategies used by other groups to achieve desired conductance levels[30,31]. The input pulses can also be modified as per the device's response to maximize linearity if required as adopted by several other investigations[32–35]. As indicated from the figures, higher magnitude of absolute conductance requires larger amplitude of erase voltages to erase the channel conductance back to the initial state and achieve symmetry. Intuitively, the large magnitude of absolute and percentage weight changes during potentiation reflects the larger number of trapped holes and hence, the larger amplitude of erase voltages adopted in turn indicates the magnitude by which the Fermi level should be raised in order to accumulate enough additional electrons in the channel to recombine with larger number of trapped holes.

In summary, the exquisite write linearity afforded by the optical gating is the major phenomenon we exploit in this work to get very accurate weights in a DRNN.

Supplementary Note-8. Neural Network Simulations

Linear Dynamic Range

To assess the benefit of the high write linearity and low write noise provided by the opto-electronic write-erase operation, we simulate several neural networks for image and speech recognition. The two parameters of linear range and write noise can be combined into one metric, linear dynamic range (LDR), defined as follows:

$$\mathrm{LDR} = \frac{\text{Range of conductance over which updates are linear}}{\sigma}$$

where σ denotes standard deviation of write noise. Intuitively, LDR points to the maximum number of states that can be available from a device for blind updates since the standard deviation of write noise sets the minimum step size. For either potentiation or depression, we denote by ΔG the step size of conductance change for application of a pulse of duration T_s (Figures-3b-j). Deviation of the measured conductance of one device in Figure-3a, Supplementary Figure-8 from a best fit straight line is very little (Supplementary Figure-17) demonstrating excellent linearity throughout the entire conductance range. Combining this with the measured write noise standard deviation (Figures-3e-j), we can calculate the LDR for our PEN as 35.4x980≈34692. Compared to other recently reported devices (Figure-4d), our PENs show at least an order of magnitude higher LDR. LDR has been calculated across 5

devices and 3 wavelengths and found to vary in the range 6311-34692 with an average value of 15102.

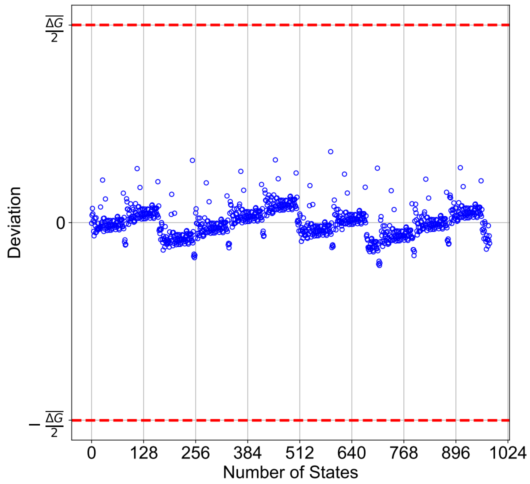

Supplementary Figure 17. Fitting a straight line to the measured conductance (Figure-3a, Supplementary Figure-8 Main Text) shows very little deviation from linearity (smaller than half of step size).

Two-shot Write Scheme

In this work, we propose to train neural networks offline and then transfer the weights by electro-optic means to the PEN crossbar for electrical inference. The advantages of this method are as follows:

- Previously reported work[36,37] using online learning for memristors could only use stochastic gradient descent (SGD) to train fully connected networks (FCN) to classify handwritten digits from the MNIST dataset. However, to train DRNN for classifying complex datasets for speech recognition, it is necessary to use sophisticated momentum based learning rules such as ADAM[38]. Hence, we propose to train the network offline and then transfer the learnt weights on the PEN array with high write accuracy.
- In-situ training of convolutional feedforward and recurrent neural networks have been recently proposed[39]. Learning in a hardware neural network can be decomposed into 3 broad phases: (1) error gradient backpropagation, (2) calculation of change in weight Δw based on backpropagated gradient and (3) physical update of weights according to the calculated Δw in step (2). While Wang et al.[39] could demonstrate that it is possible to accelerate step (1) using a resistive crossbar ideally in O(1) time, steps (2) and (3) can be done in O(1) time in-situ only if the weight update rule is stochastic gradient descent[40]. For momentum based learning rules such as RMSprop or ADAM, separate variables have to be maintained for each weight to keep

track of its weight update history. In other words, since momentum based weight update cannot be directly written as an outer product of two vectors, it cannot be performed in-situ in a memristive crossbar and requires ex-situ computation for each weight. This justifies our choice of training offline with similar requirement of ex-situ calculation of weights as well.

- ADAM has several advantages over traditional stochastic gradient descent. Firstly, it contains a momentum term which accelerates the learning process and prevents it from getting stuck at local optima. Secondly, in ADAM, the learning parameters are adapted on per-parameter basis based on previous values of parameter updates which results in smaller updates for frequent features and larger updates for infrequent features. This improvement is particularly useful for sparse gradients. Since the learning parameter adaptation is done based on only past few parameter updates, this optimization technique is also more useful than SGD for online learning and non-stationary objectives. Thirdly, since the learning parameters in ADAM are adapted on per-parameter basis, it is considered more robust towards choice of hyper parameters. Finally, ADAM calculates the bias-corrected parameter estimations which prevents it from being biased towards initial gradient and momentum values. These advantages have made ADAM the most popular choice for gradient descent optimization in past few years[41].
- Our method of weight writes involve optical stimulation, but after the write step, the inference operation is performed fully in the electrical mode, thus still enjoying high energy efficiency afforded by in-memory computing on PEN memristive arrays.

We next describe the high accuracy weight transfer scheme. From the measured data, we can estimate $\overline{\Delta G}$ and σ as the mean and standard deviation of ΔG across different trials and various initial conductance states. Based on the linearity of the conductance change, we can estimate the write pulse width T_w to reach a desired conductance G_{des} from an initial conductance G_{init} as:

$$T_{\mathrm{w}} = T_{\mathrm{s}} \times \frac{\mathrm{G_{des}} - \mathrm{G_{init}}}{\overline{\Delta \mathrm{G}}} \ldots\ldots (3)$$

where

T_w: Write pulse width to achieve desired conductance

T_s: Unit pulse width used in characterization experiments (Figures-2B-J, main text)

G_{des}: Desired final conductance

G_{init}: Initial conductance

However, it is impractical to estimate the average of ΔG with many measurements to get a best fit line for every PEN. Note that for other non-volatile memory with non-linear characteristics[42–44], it typically requires 20-30 iterations of successive write and measurement to converge to a precise conductance (<1 % error). The exquisite linearity and low write noise of the PEN allows us to simplify this procedure and reduce the number of iterations by an order of magnitude as described next.

The quickest method is to estimate the slope $\Delta G/T_P$ as difference in conductance after one write pulse of known pulse-width T_P—we refer to it as the **two-shot write scheme** where the first write is used to estimate the rate of conductance change of the PEN while the second write is used to program the PEN to the desired conductance value based on the earlier estimated slope. This is illustrated in Supplementary Figure-18 where the initial optical write (Op W) is used to increase the conductance of all devices on a chip to a very large value (greater than largest desired conductance) denoted by G_n^{op} for the nth device. Note that a global optical write is easier to implement in hardware since it does not require optical selectivity. This is followed by a measurement (m1) to read this conductance thus eliminating the mismatch across devices in optical write efficiencies. Next, the first electrical write pulse (w1) of width T_P is applied to help in slope estimation or calibration.

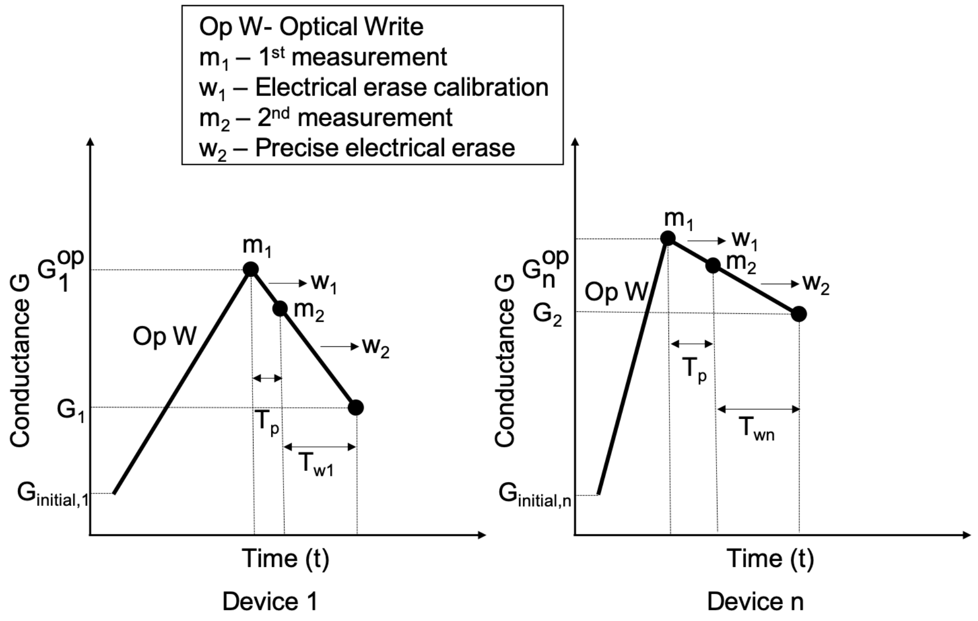

Supplementary Figure 18. Two shot write scheme for transferring learned weights to PEN crossbar. After an initial optical potentiation of the entire array, one measurement is done to estimate the conductance G_n^{op} for the nth device. Next, one electrical write (w1) operation is done for duration T_P followed by a measurement m2 to estimate change in conductance or slope. Finally, the second write pulse (w2) is applied with the duration T_{wn} calculated based on the earlier estimated slope.

The following conductance measurement (m2) allows the controller to estimate the slope for the nth device as $\Delta G_n/T_p$. Then, it can apply the second and final write pulse (w2) for the calculated width T_{wn} to reach desired final conductance following Supplementary Equation-3 but replacing the ideal slope of the best-fit line $\frac{\overline{\Delta G}}{T_s}$ with the estimate $\Delta G_n/T_p$ and using $G_{init,n}$ as the conductance of the nth device after w1.

However, this will be prone to estimation error due to write noise, measurement noise induced variability as well as any non-linearity in the device write characteristics over a large conductance range. To counter this partially, we use a long write pulse with width $T_p = nT_s$ to make this estimation (n>1). This ensures that the mean value of ΔG_n obtained after the calibration write w1 is large compared to the write noise. Also note that measurement noise is assumed negligible compared to write noise in this analysis which is reasonable since measurement circuits shared along columns can be made precise due to relaxed area/power requirements compared to elements within the crossbar.
Hence, the actual device conductance, G_{act} obtained after the second write w2 differs from G_{des} and is modelled as follows:

$$G_{\text{act}} = (1 + p) \times G_{\text{des}} + \sigma \ldots\ldots\ldots.(4)$$

where (1+p) is the slope and σ is the standard deviation or write noise calculated from actual measured device conductances.

For the best fit straight line obtained by fitting a line to the conductance of all 980 states (for the device data in Supplementary Figure-17) averaged across multiple write cycles, we get the slope error $p \approx 0$. For $T_p=16T_s$, $|p|$=0.0008 (or 0.08 %). The difference between the best-fit line and the estimated line based on the two-shot write scheme are plotted in Supplementary Figure-19a for the data corresponding to the last few states of one device. Note that the effect of write noise is minimized here by averaging the data across multiple write cycles. Here, the slope estimation is done based on the initial 16 states corresponding to $T_p = 16T_s$. It can be clearly seen that there is a systematic error in the prediction at the higher states for a straight line fit based on the initial 16 states. The error is plotted for all the states in Supplementary Figure-19b and indeed shows a distinct increasing trend with the states. Hence, it crosses the limit of half step size at an earlier point reducing the LDR to ~600 from the original number of 980 states. Both the potentiation and depression curves are modelled in this way and the

corresponding parameters are calculated. The minimum change in conductance that can be reliably obtained is limited by σ, while the effective number of bits can be obtained by the range of conductances available. To analyze the effect of different linear range of conductances, we parameterize it in terms of the number of bits n. The range of conductance (G) is [G_{min}, G_{max}] defined as:

$G_{max} = G_{min} + 2^{n} \times \overline{\Delta G}$ ……(5)

where $\overline{\Delta G}$ is the average step size and n is the number of bits.

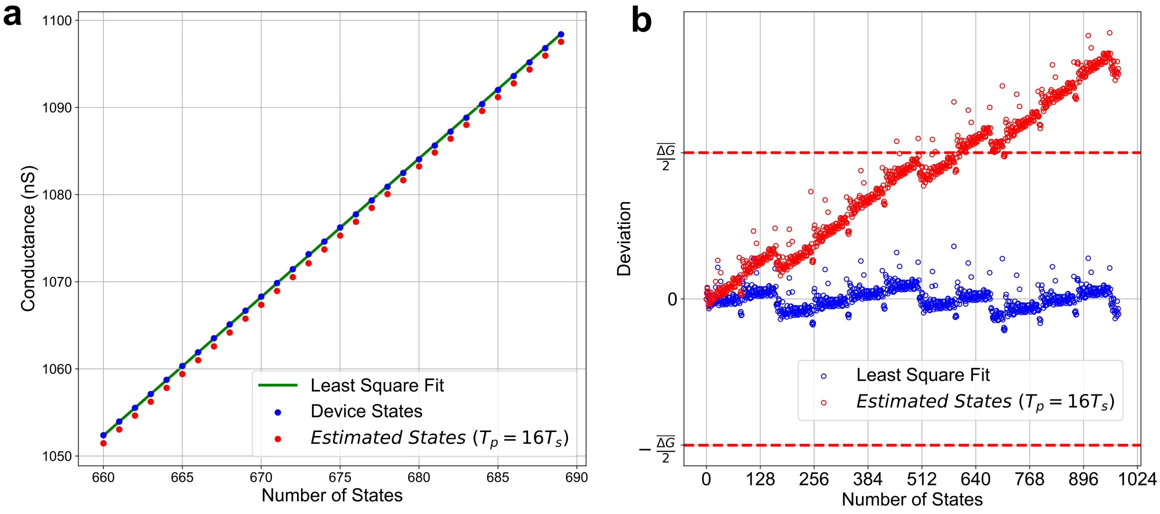

Supplementary Figure 19. a The slope estimated from the first 16 states (corresponding to $T_p = 16T_s$) results in error in predicting the conductances compared to the best fit line which measures all the states. Device data from Supplementary Figure-17 is replotted showing the difference between the best-fit line and the estimated line based on the two shot write scheme on the last few states. **b** Deviation of the conductance for all the states in Supplementary Figure-17 are replotted showing the slope estimation method results in a systematic error component that increases monotonically reducing the effective number of states within linear range.

Neural Network Simulations: MNIST and Speech classification

We simulate the case where the network is trained offline and the trained weights are written to the neuromorphic device by the earlier described two-shot write scheme. It should be noted that we can also perform online learning with SGD using blind updates as is typically shown for resistive memory crossbars trained to do handwritten digit recognition tasks based on the linearized electrical write and erase operations (Supplementary Note-5 Supplementary Figure-9). However, we focus on the results of the offline learning procedure since the focus of the paper is implementing DRNN which cannot be trained by SGD.

The procedure to map trained weights to device conductances and check the eventual accuracy in neural network simulations is as follows:

1. Train network offline to obtain trained weights ($W^{trained}$)

2. For each layer of weights, map the weight range [W_{min}, W_{max}] to [G_{min}, G_{max}] to obtain $W_{mapped}^{trained}$.

3. Read: Initial conductances (G_{init}).

4. Compute delta weights: $\Delta W = W_{mapped}^{trained} - G_{init}$

5. Compute device weights: $W^{device} = G_{init} + \text{potentiation according to max}\ (\Delta W)$ (based on eqn. 1 for potentiation curve)

6. Read: $\Delta W = W_{mapped}^{trained} - W^{device}$

7. Compute device weights: $W^{device} = W^{device} + \text{depression according to}\ \Delta W$ (based on eqn. 1 for depression curve)

8. Remap W^{device} to [W_{min}, W_{max}] to obtain $W_{remapped}^{device}$.

9. Test accuracy of the model on $W_{remapped}^{device}$.

<u>MNIST:</u>

For the experiments, we trained a CNN model[45] to classify digits from MNIST[46] dataset and a hybrid CNN+LSTM model to classify audio from tensorflow speech recognition challenge dataset[47]. The MNIST dataset consists of 28×28 pixel images of handwritten digits (0-9) from multiple writers and is relatively small compared to the speech dataset. It has been used by most earlier works to report their classification accuracy. Hence, we perform MNIST classification as a proof of concept of our two-shot write method and to enable us to compare with other earlier works as well. The CNN model was trained on 60,000 training samples to classify the 10 handwritten digits and tested on 10,000 testing samples. The network architecture used can be described as: $28 \times 28 \times 1 - 32c3 - 64c3 - m2 - 0.5d - fc128 - 0.5d - fc10$ where the input dimension is $28 \times 28 \times 1$, XcY represents a convolution layer with X convolution filters with $Y \times Y$ size, mZ represents a $Z \times Z$ 2D max-pooling layer, Pd represent a dropout layer with dropout rate P and fcL represent a fully connected dense layer with L neurons. The maximum accuracy obtained by the network for 980 states and ΔG estimation using $T_p=16T_s$ is 99.09 % (±0.1 %) which is almost same as original model accuracy (99.2 %). Supplementary Figure-20 shows the accuracy of the model with device weights ($W_{remapped}^{device}$) with varying linear dynamic range (obtained by varying the number of states). It

can be seen that for the MNIST task, even a low value of LDR (~50) is good enough to achieve high accuracy. Since the MNIST task is quite simple, it was not suitable to explore the effect of each device non-ideality separately. We next proceed to do that with the speech classification task.

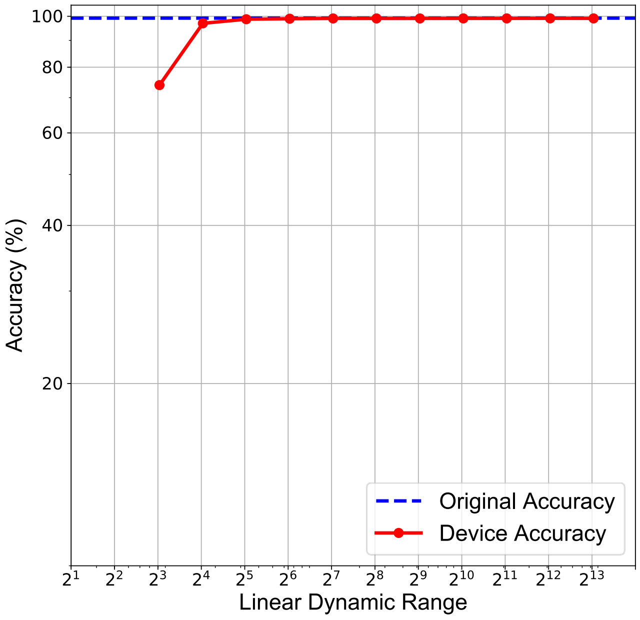

Supplementary Figure 20. Accuracy vs linear dynamic range for CNN with device weights when tested on the MNIST handwritten digit recognition task.

<u>Speech Recognition:</u>

For the Tensorflow speech recognition challenge, the dataset consists of 64,727 audio files spoken by thousands of speakers. The audio recordings are 1 sec clips of voice commands such as *'yes'*, *'no'*, *'up', 'down'* etc. The dataset was divided into 90%-10% for training and testing for 12 class classification ('yes', 'no', 'up', 'down', 'left', 'right', 'on', 'off', 'stop', 'go', 'unknown' or 'silence'). The audio clips were converted to 256×101 spectrograms and transposed spectrograms were given as input to the hybrid CNN-RNN model. The architecture of the network can be described using aforementioned notations as: $256 \times 101 \times 1 - bn - 64c(5,7) - 64c3 - m3 - 64c3 - 64c3 - m(2,3) - 128c3 - 128c3 - m(2,3) - m(2,3) - 128c3 - 128c3 - bl256 - 0.5d - fc200 - 0.5d - fc12$ where bn is batch normalization layer, *bl* is Bidirectional LSTM layer and *Xc(Y, Z)* represents a convolution layer with *X* convolution filters with $Y \times Z$ size. The 3.62 million trainable weights in this model is an order of magnitude higher than other simulated networks using resistive memory device to

classify handwritten digits (Figure-4d). The software model of our proposed network with floating point weights achieved an accuracy of 95.71% on the word recognition task.

From the accuracy plots of the word recognition task (Figure-4c) we can conclude that relatively larger LDR (~256) is required for word recognition compared to the MNIST task. We hypothesize that this is because of the recurrent neural layers (LSTM) in the speech recognition network which have the highest number of parameters. Also, any errors in mapping weights may be magnified by the recurrence. To test this hypothesis, we perform three experiments where only the convolutional layer, fully or densely connected layer or the recurrent LSTM layers are quantized using linear quantization. For the convolutional and fully connected layers, those with the largest number of parameters are chosen. The results plotted in Figure-4c indeed show that the accuracy of the LSTM layer is most sensitive to bit precision and drops the earliest when LDR reduces.

Finally, we explored the effect of reduced estimation error during weight mapping by taking larger estimation times as well as using the best fit straight line to estimate the conductances. As can be seen in Supplementary Figure-21, the classification accuracy of ~93.5 % drops by around 30 % when the slope estimation time is reduced by half from $16T_s$ to $8T_s$. The best case corresponds to a least square fit line through all the 980 conductance states. In that case, we obtain back the original accuracy of 95.7 % similar to using floating-point numbers. This confirms that the slope estimation errors are the major limiting factor in achieving higher accuracy.

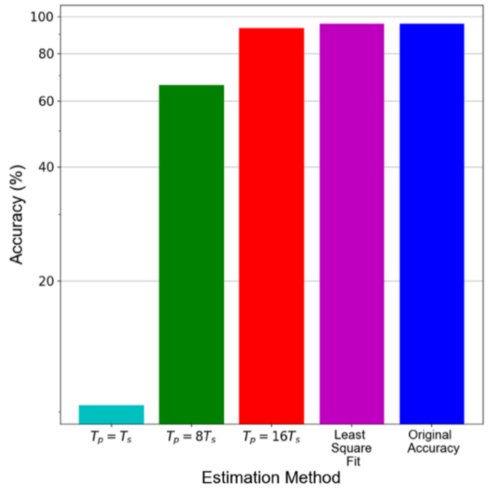

Supplementary Figure 21. Reducing conductance slope estimation error by increasing measurement time results in same accuracy as floating-point implementation when the least square fit line using all 980 states is used. Using

only 16 points to estimate the slope, results in ~ 2 % drop in accuracy while using 8 points result in ~30 % drop in accuracy.

Supplementary Note-9. Extending this concept to other material platforms

The proposed optoelectronic architecture can be generalized to a wide variety of semiconducting platforms (i.e., III-V semiconductors, transition metal dichalcogenides (TMDCs), halide perovskites and organic semiconductors), opening up new possibilities with improved scalability and CMOS compatibility.

To prove the universal nature of the proposed concept, additional experiments were carried out on Poly(3-hexylthiophene) (P3HT) semiconducting films in a thin film transistor configuration. Supplementary Figure-22 below depicts the linear weight updates in a P3HT memtransistor configuration. Optical pulses enabled programming or writing of conductance states, while electrical voltages to the back-gate acted as erasing pulses. The source and drain electrodes acted as the reading terminals across which the memconductance or memresistance was read. As evident from the graph below, weight updates with excellent linearity could be programmed facilely via optical pulses. By optimising the light intensity and pulse width, it was possible to access a large number of non-volatile states (~440) with excellent conductance linearity and low write noise.

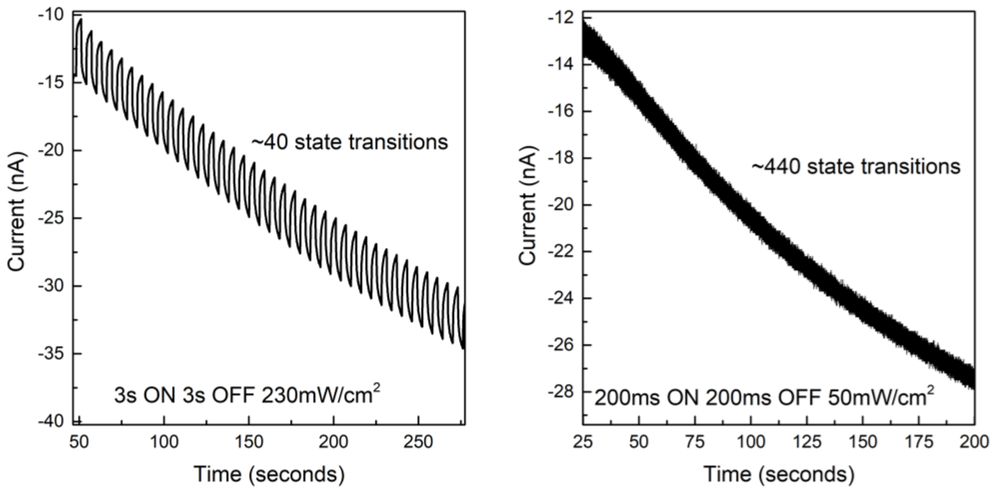

Supplementary Figure-22. Optically-addressed P3HT multi-level memory for DRNNs. These devices were subjected to an input optical pulse train of constant pulse width and interval (λ=445 nm). By optimising the light intensity and pulse width, it was possible to access a large number of non-volatile states with excellent conductance linearity and low write noise.

To demonstrate the universality of our programming approach and develop a general mechanistic understanding, further experiments were conducted on Molybdenum disulphide (MoS_2), Black Phosphorus (BP) and Poly(3-hexylthiophene) (P3HT) PENs as detailed below.

Effect of constant gate bias V_{gs}:

We observe that the linearity can be modulated in all the above systems as a function of the applied constant gate bias during optical potentiation, similar to ReS_2 FETs (Supplementary Figure-23). Please note that MoS_2 FETs are n-type and hence requires a large negative gate voltage to keeps the trap states empty, while p-type BP and P3HT FETs require a high positive gate voltage for the same. In the case of P3HT PENs, the average retention of the non-volatile states is <200 s as supposed to >900 s for our 2D TMDC FETs.

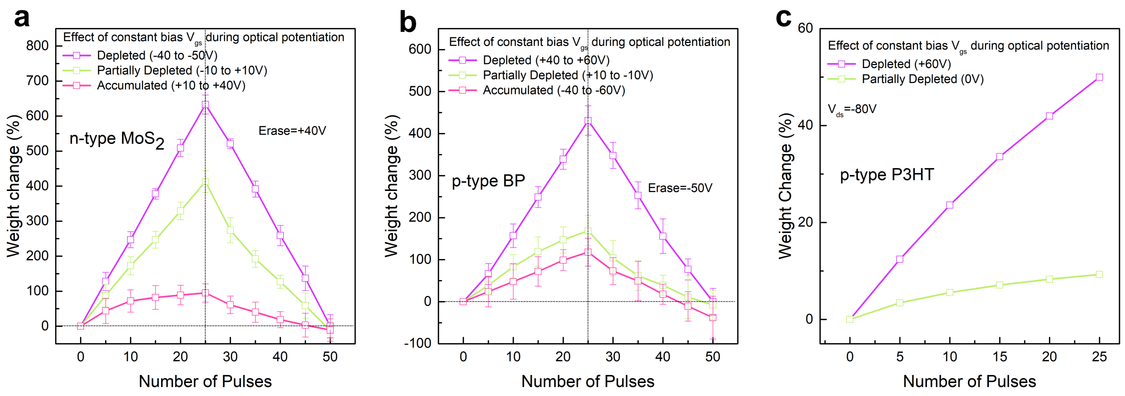

Supplementary Figure 23. a Weight changes in **a** n-type MoS_2 [Data from 15 devices], **b p**-type BP PENs [Data from 5 devices] and **c** p-type P3HT OFETs as a function of the constant bias V_{gs}. For potentiation, blue light pulses of λ=445 nm and intensity=65 mWcm^{-2} was used with a pulse width and interval of 10 s each for MoS_2 and BP, while 23 mWcm^{-2} together with a pulse width and interval of 2 s each was used for P3HT. Key for MoS_2: Depleted- refers to a range of V_{gs} -40 to -50 V depending on the V_{on} of the respective PEN. Similarly, Partially Depleted- refers to a range of V_{gs} -10 to +10 V and Accumulated- refers to a range of V_{gs} +10 to +40 V. Key for BP: Depleted- refers to a range of V_{gs} +40 to +60 V depending on the V_{on} of the respective PEN. Similarly, Partially Depleted- refers to a range of V_{gs} +10 to -10 V and Accumulated- refers to a range of V_{gs} -40 to -60 V. Key for P3HT: Depleted- refers to a of V_{gs} +60 V and Partially Depleted- refers to a of V_{gs} 0 V. The graph represents our conclusion from experimental measurement of 15 MoS_2, 5 BP and P3HT FETs. The error bars represent the variation among devices.

The dependence of background conductance and illumination intensity of the devices on linearity was confirmed with studies on MoS_2 PENs as shown below (Supplementary Figure-24).

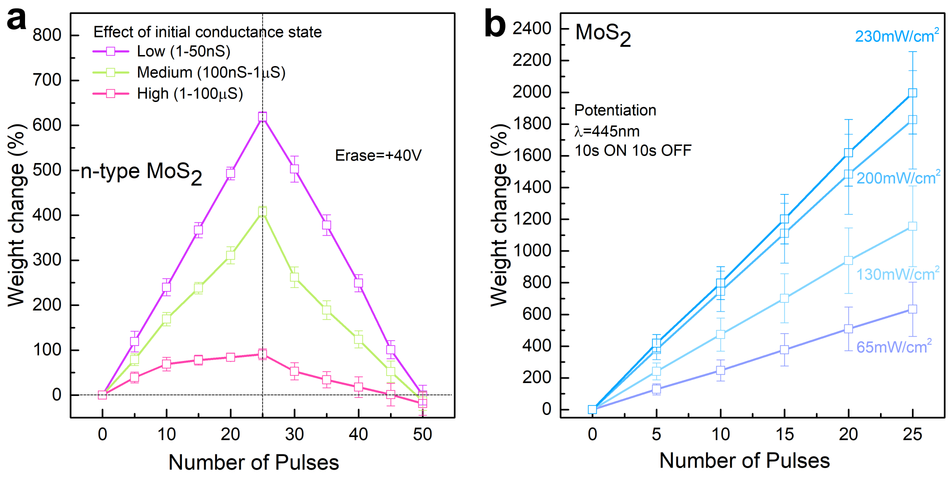

Supplementary Figure 24. a Weight changes in MoS_2 PENs [Data from 15 devices] as a function of the initial conductance state. Key: Low- refers to a range of 1-50 nS depending on the respective PEN. Similarly, Medium- refers to a range 100 nS-1 μS and High- refers to a range of 1-100 μS. The initial conductance here refers to the conductance of the FET before potentiation and depression measurements. For a fair comparison, the same fully depleting voltage (V_{gs}=-40 V) was applied to all devices. **b Weight changes as a function of the light intensity** for λ= 445 nm, 10 s ON, 10 s OFF in MoS_2 PENs [Data from 5 devices]. The graph represents our conclusion from experimental measurement of 5 MoS_2 FETs. The error bars represent the variation among devices.

Effect of drain bias V_{ds}:

We next investigate the effect of V_{ds} in P3HT FETs by comparing the weight change responses under the same optical pulsing conditions and V_{gs} bias (Supplementary Figure-25). As mentioned in Supplementary Note-7, a V_{ds} that ensures the channel currents are much larger that the leakage currents is necessary for accurate weight read-outs. Interference of the gate leakage currents affect the read outs and their linearity and hence, is a very critical requirement[15,28]. For the P3HT FETs, a high drain voltage of -80 V is required to extract excellent linearly varying photo-memory effects. Low V_{ds} result in poor margins between I_{ds} and I_{gs} as evident from their transfer characteristics, impairing the extraction of linear optical weight updates. As shown in the figure below, at V_{ds}= -1 V, the gate leakage current I_{gs} (10^{-8} A) is much larger than the drain current I_{ds} (10^{-9} A), preventing reliable weight readouts. At -5 V, I_{ds} is larger than I_{gs}, but with very low margin (<3x). At -60 V and higher, I_{ds} becomes much larger than I_{gs} with good readout margins (>200x), allowing accurate weight readouts with very high signal to noise ratio. Please note that the magnitude of normalized weight changes at high drain voltages are lower than those at low drain voltages in the figure below. However, since

the channel conductance at V_{ds}=-5 V is heavily influenced by the gate leakage currents (refer to the I_dV_g curves on the right), we believe that only the weights extracted from V_{ds}=-80 V reflect accurate read outs.

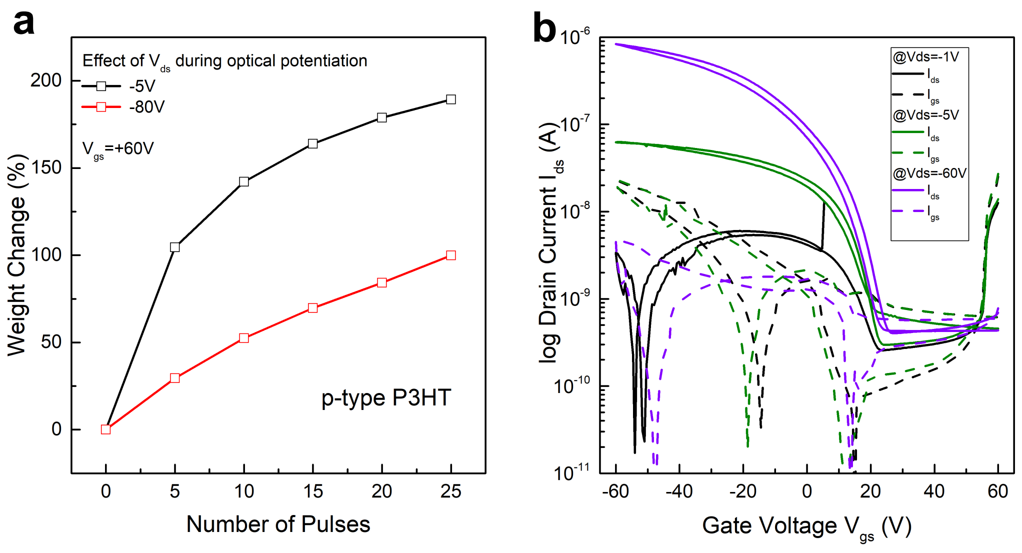

Supplementary Figure 25. a Weight changes in p-type P3HT OFETs as a function of V_{ds}. For potentiation, blue light pulses of λ=445 nm and intensity=23 mWcm^{-2} was used with a pulse width and interval of 2 s each. V_{gs} was held constantly at +60 V (fully depleted mode) throughout the measurements. **b** Transfer characteristics of P3HT OFETs under different V_{ds}.

Supplementary References:

1. Wang, Q. H., Kalantar-Zadeh, K., Kis, A., Coleman, J. N. & Strano, M. S. Electronics and optoelectronics of two-dimensional transition metal dichalcogenides. *Nat. Nanotechnol.* **7**, 699 (2012).
2. John, R. A. *et al.* Synergistic Gating of Electro-Iono-Photoactive 2D Chalcogenide Neuristors: Coexistence of Hebbian and Homeostatic Synaptic Metaplasticity. *Adv. Mater.* **30**, 1800220 (2018).
3. Arnold, A. J. *et al.* Mimicking neurotransmitter release in chemical synapses via hysteresis engineering in MoS2 transistors. *ACS Nano* **11**, 3110–3118 (2017).
4. Abbott, L. F. & Nelson, S. B. Synaptic plasticity: taming the beast. *Nat. Neurosci.* **3**, 1178–1183 (2000).
5. John, R. A. *et al.* Flexible Ionic-Electronic Hybrid Oxide Synaptic TFTs with Programmable Dynamic Plasticity for Brain-Inspired Neuromorphic Computing. *Small* **13**, (2017).
6. Wu, S., Wong, K. Y. & Tsodyks, M. Neural information processing with dynamical

synapses. *Front. Comput. Neurosci.* **7**, 188 (2013).

7. Tiwari, N. *et al.* Indium tungsten oxide thin films for flexible high-performance transistors and neuromorphic electronics. *ACS Appl. Mater. Interfaces* **10**, 30506–30513 (2018).
8. Sjostrom, P. J., Rancz, E. A., Roth, A. & Hausser, M. Dendritic excitability and synaptic plasticity. *Physiol. Rev.* **88**, 769–840 (2008).
9. Song, S., Miller, K. D. & Abbott, L. F. Competitive Hebbian learning through spike-timing-dependent synaptic plasticity. *Nat. Neurosci.* **3**, 919–926 (2000).
10. Caporale, N. & Dan, Y. Spike timing–dependent plasticity: a Hebbian learning rule. *Annu. Rev. Neurosci.* **31**, 25–46 (2008).
11. John, R. A. *et al.* Ultralow Power Dual-Gated Subthreshold Oxide Neuristors: An Enabler for Higher Order Neuronal Temporal Correlations. *ACS Nano* **12**, 11263–11273 (2018).
12. John, R. A. *et al.* Ionotronic Halide Perovskite Drift-Diffusive Synapses for Low-Power Neuromorphic Computation. *Adv. Mater.* 30,1805454 (2018).
13. Kulkarni, M. R. *et al.* Field-Driven Athermal Activation of Amorphous Metal Oxide Semiconductors for Flexible Programmable Logic Circuits and Neuromorphic Electronics. *Small* 15,1901457 (2019).
14. Fuller, E. J. *et al.* Parallel programming of an ionic floating-gate memory array for scalable neuromorphic computing. *Science.* **364**, 570–574 (2019).
15. Leydecker, T. *et al.* Flexible non-volatile optical memory thin-film transistor device with over 256 distinct levels based on an organic bicomponent blend. *Nat. Nanotechnol.* **11**, 769–775 (2016).
16. Fuller, E. J. *et al.* Li-ion synaptic transistor for low power analog computing. *Adv. Mater.* **29**, (2016).
17. Keene, S. T. *et al.* Optimized pulsed write schemes improve linearity and write speed for low-power organic neuromorphic devices. *J. Phys. D. Appl. Phys.* **51**, 224002 (2018).
18. Cho, K. *et al.* Electric stress-induced threshold voltage instability of multilayer MoS2 field effect transistors. *ACS Nano* **7**, 7751–7758 (2013).
19. Late, D. J., Liu, B., Matte, H. S. S. R., Dravid, V. P. & Rao, C. N. R. Hysteresis in single-layer MoS2 field effect transistors. *ACS Nano* **6**, 5635–5641 (2012).
20. Li, T., Du, G., Zhang, B. & Zeng, Z. Scaling behavior of hysteresis in multilayer MoS2 field effect transistors. *Appl. Phys. Lett.* **105**, 93107 (2014).

21. Guo, Y. *et al.* Charge trapping at the MoS2-SiO2 interface and its effects on the characteristics of MoS2 metal-oxide-semiconductor field effect transistors. *Appl. Phys. Lett.* **106**, 103109 (2015).
22. Park, Y., Baac, H. W., Heo, J. & Yoo, G. Thermally activated trap charges responsible for hysteresis in multilayer MoS2 field-effect transistors. *Appl. Phys. Lett.* **108**, 83102 (2016).
23. Zhang, X. *et al.* Poly (4-styrenesulfonate)-induced sulfur vacancy self-healing strategy for monolayer MoS 2 homojunction photodiode. *Nat. Commun.* **8**, 1–8 (2017).
24. Zhang, S. *et al.* Defect structure of localized excitons in a WSe 2 monolayer. *Phys. Rev. Lett.* **119**, 46101 (2017).
25. Lopez-Sanchez, O., Lembke, D., Kayci, M., Radenovic, A. & Kis, A. Ultrasensitive photodetectors based on monolayer MoS 2. *Nat. Nanotechnol.* **8**, 497–501 (2013).
26. Thakar, K. *et al.* Multilayer ReS2 photodetectors with gate tunability for high responsivity and high-speed applications. *ACS Appl. Mater. Interfaces* **10**, 36512–36522 (2018).
27. Lv, Z. *et al.* Mimicking neuroplasticity in a hybrid biopolymer transistor by dual modes modulation. *Adv. Funct. Mater.* **29**, 1902374 (2019).
28. Lee, J. *et al.* Monolayer optical memory cells based on artificial trap-mediated charge storage and release. *Nat. Commun.* **8**, 1–8 (2017).
29. Jeon, S. *et al.* Gated three-terminal device architecture to eliminate persistent photoconductivity in oxide semiconductor photosensor arrays. *Nat. Mater.* **11**, 301–305 (2012).
30. Wang, Q. *et al.* Nonvolatile infrared memory in MoS2/PbS van der Waals heterostructures. *Sci. Adv.* **4**, eaap7916 (2018).
31. Yeats, A. L. *et al.* Persistent optical gating of a topological insulator. *Sci. Adv.* **1**, e1500640 (2015).
32. Seo, S. *et al.* Artificial optic-neural synapse for colored and color-mixed pattern recognition. *Nat. Commun.* **9**, 1–8 (2018).
33. Wang, I.-T., Chang, C.-C., Chiu, L.-W., Chou, T. & Hou, T.-H. 3D Ta/TaO x/TiO2/Ti synaptic array and linearity tuning of weight update for hardware neural network applications. *Nanotechnology* **27**, 365204 (2016).
34. Chen, P.-Y. *et al.* Mitigating effects of non-ideal synaptic device characteristics for on-chip learning. in *2015 IEEE/ACM International Conference on Computer-Aided Design (ICCAD)* 194–199 (IEEE, 2015).

35. Moon, K., Kwak, M., Park, J., Lee, D. & Hwang, H. Improved conductance linearity and conductance ratio of 1T2R synapse device for neuromorphic systems. *IEEE Electron Device Lett.* **38**, 1023–1026 (2017).
36. Fuller, E. J. *et al.* Li-ion synaptic transistor for low power analog computing. *Adv. Mater.* **29**, (2017).
37. van de Burgt, Y. *et al.* A non-volatile organic electrochemical device as a low-voltage artificial synapse for neuromorphic computing. *Nat. Mater.* **16**, 414–418 (2017).
38. Kingma, D. P. & Ba, J. Adam: A method for stochastic optimization. Preprint at *arXiv1412.6980* (2014).
39. Wang, Z. *et al.* In situ training of feed-forward and recurrent convolutional memristor networks. *Nat. Mach. Intell.* **1**, 434–442 (2019).
40. Agarwal, S. *et al.* Energy scaling advantages of resistive memory crossbar based computation and its application to sparse coding. *Front. Neurosci.* **9**, 484 (2016).
41. Karparthy, A. A peek at trends in machine learning. https://medium.com/@karpathy/a-peek-at-trends-in-machine-learning-ab8a1085a106 (2017).
42. Basu, A. & Hasler, P. E. A fully integrated architecture for fast and accurate programming of floating gates over six decades of current. *IEEE Trans. Very Large Scale Integr. Syst.* **19**, 953–962 (2010).
43. Basu, A. *et al.* A floating-gate-based field-programmable analog array. *IEEE J. Solid-State Circuits* **45**, 1781–1794 (2010).
44. Bayat, F. M. *et al.* Model-based high-precision tuning of NOR flash memory cells for analog computing applications. in *2016 74th Annual Device Research Conference (DRC)* 1–2 (IEEE, 2016).
45. Chollet, F. MNIST CNN- Keras Documentation. https://keras.io/examples/mnist_cnn/ (2015).
46. LeCun, Y., Bottou, L., Bengio, Y. & Haffner, P. Gradient-based learning applied to document recognition. *Proc. IEEE* **86**, 2278–2324 (1998).
47. Warden, P. Speech commands: A public dataset for single-word speech recognition. *Dataset available from http://download. tensorflow. org/data/speech_commands_v0* **1**, (2017).
